# Danon disease in male patients: a prospective natural history study to augment understanding of the phenotype

**DOI:** 10.1186/s13023-025-04058-8

**Published:** 2025-10-21

**Authors:** Tarek Khedro, Jennifer Attias, Emily Eshraghian, Melina Tsotras, Emily Margolin, Shaden Yassin, Elizabeth Silver, Quan Bui, Shyamanga Borooah, Chamindra G. Laverty, Matthew R. G. Taylor, Eric D. Adler, Kimberly N. Hong

**Affiliations:** 1 Department of Internal Medicine, Pennsylvania Hospital of the University of Pennsylvania Health System, Philadelphia, PA USA; 2 Department of Cardiology, University of California San Diego, 9500 Gilman Drive, La Jolla, San Diego, CA 92093 USA; 3 Department of Ophthalmology, University of California San Diego, San Diego, CA USA; 4 Department of Neurology, School of Medicine, University of California San Diego, San Diego, CA USA; 5 Adult Medical Genetics Program, University of Colorado, Anschutz Medical Campus, Aurora, CO USA

**Keywords:** Danon disease, Nonischemic genetic cardiomyopathy, Lysosomal-associated membrane protein 2 (LAMP2), Pediatric quality of life inventory (PQLQ), Pediatric cardiac quality of life inventory (PCQLI), Differential ability Scales-II (DAS-II), Vineland adaptive behavior scales-3 (VABS-3), North star ambulatory assessment (NSAA)

## Abstract

**Background:**

Danon disease (DD) is a rare X-linked cardioskeletal myopathy caused by pathogenic mutations in lysosomal-associated membrane protein-2 (*LAMP2*). It is a multisystemic disease that affects the heart, skeletal, neurologic and ophthalmic systems. With an early age of onset especially in males and no treatment for the cardiomyopathy outside of heart transplant, natural history studies have been paramount to providing clinical data for the development and advancement of disease-focused therapies. Herein we present a comprehensive, prospective study detailing both cardiac and extracardiac features of DD.

**Methods:**

The cohort was comprised of 8 male pediatric and 1 male young adult patient, enrolled at the University of California, San Diego. Diagnosis of DD was confirmed with a pathogenic or likely pathogenic *LAMP2* variants. The patients underwent serial quality of life, neuropsychological, cognitive, ophthalmological, cardiac, pulmonary, and neuromuscular assessments every 6 months for 3 years.

**Results:**

The mean age of the cohort at study enrollment was 11.6 ± 4.5 years. The mean age of the cohort at diagnosis was 6.5 ± 5.2 years. Quality of life: assessed through the Pediatric Cardiac Quality of Life Inventory (PCQLI) and Pediatric Quality of Life Inventory (PQLQ) reveals perceptions of poor quality of life by both patients and parents. Cognitive: Differential Ability Scales-II (DAS-II) and Vineland Adaptive Behavior Scales-3 (VABS-3) showed marked intellectual disability at baseline. Cardiac: over time, ejection fraction decreased, myocardial walls thickened, and left ventricular mass increased. Pulmonary: FVC increased over time; cardiopulmonary exercise testing (CPET) revealed decreased exercise capacity as measured by peak oxygen uptake (peak VO_2_ max) and 6-minute walk test (6MWT). Neuromuscular: most patients in the cohort achieved maximum scores on North Star Ambulatory Assessment (NSAA), with minimal changes over 6-month follow-up; they were faster than a study of normal children during the 10-meter walk test (10MWT); they were slower than a study of normal children during time to rise and the 4-stair climb test.

**Conclusion:**

Natural history studies provide an important opportunity to study rare diseases, especially when longitudinal data is available to characterize clinical changes and disease course. In the case of DD, young, male patients were found to have general deficits across quality-of-life indices and cognitive, cardiac, neuromuscular, ophthalmologic, and pulmonary functions. Stabilization or improvements in these domains may be appropriate outcomes for clinical trials to consider.

**Supplementary Information:**

The online version contains supplementary material available at 10.1186/s13023-025-04058-8.

## Background/introduction

Danon disease (DD) is a rare X-linked dominant cardioskeletal myopathy caused by pathogenic variants in the lysosomal-associated membrane protein-2 (*LAMP2*) gene—a critical regulator of autophagic flux expressed in all multicellular organisms [1]. It is classically characterized by severe cardiac hypertrophy, skeletal myopathy, and intellectual disability, but other systemic manifestations have been reported, including deficits, gastrointestinal tract issues, and neuropsychiatric disorders. In female patients, the disease is typically milder, where often only the cardiac system is affected––however, the cardiomyopathy can be as severe as in male patients.

Until recently, DD was reported to be extremely rare, with one publication indicating only 500 cases being identified since 1981 [2]. However, with the advent of next-generation sequencing a higher prevalence of DD in disease specific gene panels has been identified: up to 4–6% in children with hypertrophic cardiomyopathy (HCM) [3–5] and 0.7-4% in adults with HCM [6–8]. Notably, cardiac involvement and symptoms are near universal in all male DD patients, with the typical onset of cardiac symptoms in infancy or adolescence [2]. The extracardiac manifestations of DD remain underexplored in the current literature.

Natural history studies help characterize disease course, allowing for identification of clinical parameters that suggest progressive disease and poor outcomes. To this end, they play a pivotal role in generating data essential for supporting the formulation and refinement of clinical trial eligibility criteria and outcomes specific to rare diseases. The multisystemic longitudinal data captured in this natural history study provide a more comprehensive characterization of the clinical manifestations and progression of DD than previously reported.

In this natural history study, we studied a cohort of nine male patients with genetically confirmed DD. Previous studies on DD have been cross-sectional, whereas the current study is the largest prospective cohort with longitudinal data. The aims of our study were to describe the natural history of DD along multiple dimensions, including patient-reported outcomes, cognitive, neuromuscular, ophthalmologic, pulmonary, and cardiac testing. Our objective is to provide a guide to baseline disease characteristics and progression that clinicians and researchers can use to inform medical decision-making, including escalation of therapy and referral for transplant, and clinical trial design, respectively [9].

## Methods

### Participants and study design

We performed a single-center, prospective, cohort study, in which participation was open to all children and adults diagnosed with DD. The diagnosis was confirmed by genetic testing indicating a pathogenic or likely pathogenic variant in *LAMP2*.

All patients were enrolled at the University of California, San Diego (UCSD). Medical history, medications and clinical events were collected for the duration of the study period. Additionally, diagnostics administered longitudinally included: patient reported outcomes, ophthalmological tests, cardiac testing, neurological and neuropsychological testing, pulmonary function testing, genetic testing, and laboratory testing. Lab testing was obtained at 3-month intervals and all other testing was conducted at 6-month intervals, with a total study period of 3 years. All data collected was entered into a REDCap database administered by UCSD. Due to small sample sizes, summarized values are presented as a mean with a standard deviation. For left ventricular structural variables including ejection fraction, mass and wall thickness, and cardiac biomarkers including NT-proBNP and high sensitivity troponin T, paired comparisons overtime were made using a Wilcoxon signed rank test. Patient 7 received a heart transplant and patients 6 and 8 were enrolled in a Phase 1 gene therapy clinical trial, so data was censored after transplant and clinical trial enrollment respectively.

### Patient reported outcome measures

#### Pediatric cardiac quality of life inventory (PCQLI)

The PCQLI is intended for ages 8–18 and includes self-respondent and parent/proxy reporting and consists of two subscales (disease impact and psychosocial impact) that are combined to yield a total score [10]. The PCQLI is further subdivided by age of patient due to the language required for understanding the questions (child: 8–12 years, adolescent: 12–18 years). There was one patient under eight during visits where neither instrument was completed. Response options range from excellent to poor on a five-point Likert scale, which are converted to a four-point absolute score, and then used to calculate the Disease Impact and Psychosocial Impact subscale scores (maximum score: 50 each). The following formula is used: [(Σ subscale item response values – Number of subscale items)/(4 x Number of subscale items)]x 50. The Total Score is the sum of both of these scores (maximum score: 100). A higher score on the PCQLI is correlated with a higher quality of life.

#### Pediatric quality of life questionnaire (PQLQ)

The PQLQ includes both self-respondent and parent/proxy reporting and consists of 23 questions comprising four dimensions (physical functioning, emotional functioning, social functioning, school functioning) to yield a psychosocial health summary score, a physical health summary score, and the total score [11]. It has been validated in healthy children [12] and in those with hypertrophic cardiomyopathy [13, 14].

The PQLQ generic core scales also includes both self-respondent and parent/proxy reporting and is further subdivided by age of patient: young children (ages 5–7), children (ages 8–12), and teens (13–18). Items are answered on a five-point Likert scale from 0 (not at all) to 4 (almost always). Items are reversed scored and linearly transformed to a 0-100 scale (0 = 100, 1 = 75, 2 = 50, 3 = 25, 4 = 0). Additionally, three summary scores are calculated: psychosocial health summary score (average score of items across the emotional, social, and school functioning domains), physical health summary score (only composed of the physical functioning score, and therefore equal), and a total score (average of items across all domains). Total scores range from 0 to 100, with higher scores indicating better quality of life.

### Neuropsychological assessment

For the current study, two cognitive and behavioral assessments were performed: the DAS-II and VABS-3.

The DAS-II is a cognitive assessment instrument that assesses both general cognitive ability and verbal, nonverbal, and spatial reasoning. The DAS-II produces a General Conceptual Ability (GCA) score corresponding to full scale IQ, as well as standardized scores in verbal, nonverbal, and spatial domains [15].

The VABS-3 is a cognitive assessment used to examine adaptive behavior, specifically an individual’s proficiency in daily living skills necessary for personal and social sufficiency [16]. Individuals are scored on four domains: the Adaptive Behavior Composite (ABC), communication, daily living skills, and socialization [17]. It evaluates how well an individual can function in everyday life and correlates with composite and verbal IQ scores but not with nonverbal or spatial scores [17]. In clinical and educational settings, the VABS assesses adaptive functioning, diagnosing developmental disabilities, and guiding intervention planning [16]. Here, the items are graded based on the frequency with which the individual performs each skill, with higher numbers reflecting stronger adaptive behavior. Both assessments were administered and graded by a licensed psychologist who specialized in pediatric neuropsychology.

### Ophthalmological assessments

An ophthalmological examination was performed which included an assessment of best-corrected visual acuity (BCVA) using a Snellen chart, slit lamp biomicroscopy, and ophthalmoscopy. Multimodal imaging was also performed, which included Spectral Domain Optical Coherence Tomography (SD-OCT) (Heidelberg Engineering, Heidelberg, Germany, Software Version: 6.3.4), ultra-wide-field pseudocolor photos, and fundus autofluorescence (FAF) (Optos, Nikon, UK).

### Cardiac assessment

All patients had a cardiac evaluation performed, which consisted of clinical symptoms, New York Heart Association (NYHA) Classification, electrocardiographic and echocardiographic parameters including left ventricular end diastolic dimension (LVEDD), global longitudinal strain (GLS), maximal wall thickness (MWT), ejection fraction (EF), and LV mass.

### Cardiac and pulmonary functional assessments

Pulmonary function tests (PFTs) included spirometry and lung volumes. Specific parameters that were measured included: upright and supine forced vital capacity (FVC), forced expiratory volume (FEV1), and FEV1/FVC. Cardiopulmonary exercise testing (CPET) was also completed via treadmill ergometry. Peak oxygen consumption (VO_2_ max) (mL/kg/min), an integrated measure that reflects pulmonary and cardiovascular extraction and delivery of oxygen, cellular utilization of oxygen, and ventilatory efficiency (V_E_/VCO_2_ slope) were reported.

### Neuromuscular assessment

North Star Ambulatory Assessment (NSAA) was completed for each patient at each visit. NSAA score captures information on neuromuscular and motor function and physical performance and includes seventeen functional motor abilities testing, each of which is scored on a three-point scale; 2 = normal, achieves goal without any assistance; 1 = modified method but achieves goal independent of physical assistance from another person; 0 = unable to achieve independently [18]. Total score is achieved by summing the scores for all the individual items and ranges from 0, if all activities are failed, to 34, if all activities are achieved. The NSAA also includes two record timed items (10-meter timed walk/run test [10MWT] and time to rise from the floor or Gower test) [19]. The 6-minute walking distance test (6MWT) provides data on distance walked, time taken, and the use of walking aids. The 4-stair climb (4SC) test was also completed.

## Results

### Visit completion rates

Nine male patients were enrolled during January 2019 to September 2019. Due to the COVID-19 pandemic, study participation was incomplete. Pre-COVID pandemic onset (March 2020), 80.1% of the expected 171 visits were attended (including baseline visits). Post-COVID pandemic, 19.7% of the 458 scheduled visits were attended. The cognitive assessments DAS-II and VABS-3 were performed at baseline only. All other assessments had follow-up visits completed at varying time points (Supplementary Table 1).

### Study population and genetic analysis results

We included eight pediatric males and one adult male from the USA, comprising eight families. The characteristics of the study population are summarized in Table 1. Nonsense mutations occurred in six patients (66%) and three patients (33%) had deletions (Table 2).

**Table 1:** Characteristics of the study cohort, *n* = 9

Demographics	*n*	Percentage
Male	9	100%
**Ethnicity**		
Non-Hispanic/Latin	9	100%
**Race**		
White	8	89%
Asian	1	11%
**Index or Family**		
Index case	5	56%
Family member of index	4	44%
**De Novo Mutation**		
Yes	4	44%
No	5	56%
**Mutation site**		
Exon	9	100%
**Mutation type**		
Nonsense	6	67%
Deletion	2	22%
Frameshift	1	11%
**Pathogenicity**		
Pathogenic	5	56%
Likely pathogenic	4	44%
**Ambulatory**		
Yes	9	100%
**Characteristics**		
Mean age at diagnosis, years	**6.5 ± 5.2**	
Mean age at enrollment, years	**11.6 ± 4.5**	
Mean height, cm	**151.3 ± 26.2**	
Mean weight, kg	**54.3 ± 25.3**	

Two of the patients in the cohort are brothers

**Table 2:** Genetic screening results for the nine patients

ID	DNA sequence change	Amino acid change	Site of mutation	Type
1	NM_002294.3:c.718 C > T	p.Gln240*	Exon 5	Nonsense
2	NM_001122606.9:Xq24del119580840-119581776		Exon 4 to 6	Deletion
3	NM_002294.2:Xq24del119333981-119620455)	Entire protein	Entire gene	Deletion
4	NM_002294.2:c.109G > C	p.Glu37*	Exon 9	Nonsense
5	NM_002294.2:c.109G > C	p.Glu37*	Exon 9	Nonsense
6	NM_002294.2:c.1219delA		Exon 8	Frameshift
7	NM_002294.2:c.877 C > T	p.Arg293*	Exon 7	Nonsense
8	NM_002294.3 c.741G > A	p.Lys247=	Exon 5 (last base pair/splice region)	Nonsense
9	NM_002294.2:c.877 C > T	p.Arg293*	Exon 7	Nonsense

Patients 4 and 5 are brothers

### Patient reported outcome measures

#### PCQLI

##### Patient responses

Seven of eight pediatric patients had baseline PCQLI responses (88%, *n* = 7), of which seven had follow-up responses (100%, *n* = 7). The baseline mean for the Disease Impact subscale was 25.6 ± 6.0. The mean follow-up time was 26.6 months for both patient and parent responses (see all responses in Supplementary Table 2). At last follow up, four patients (57%, *n* = 4) reported lower Disease Impact scores. For the Psychosocial Impact Score, the mean at baseline was 28.9 ± 7.5. At last follow-up, three patients (43%, *n* = 3) reported lower scores. For the Total Score, the mean was 54.5 ± 10.5 at baseline. At last follow-up, four patients (57%, *n* = 4) reported lower total scores.

##### Parent responses

**Fig. 1:**
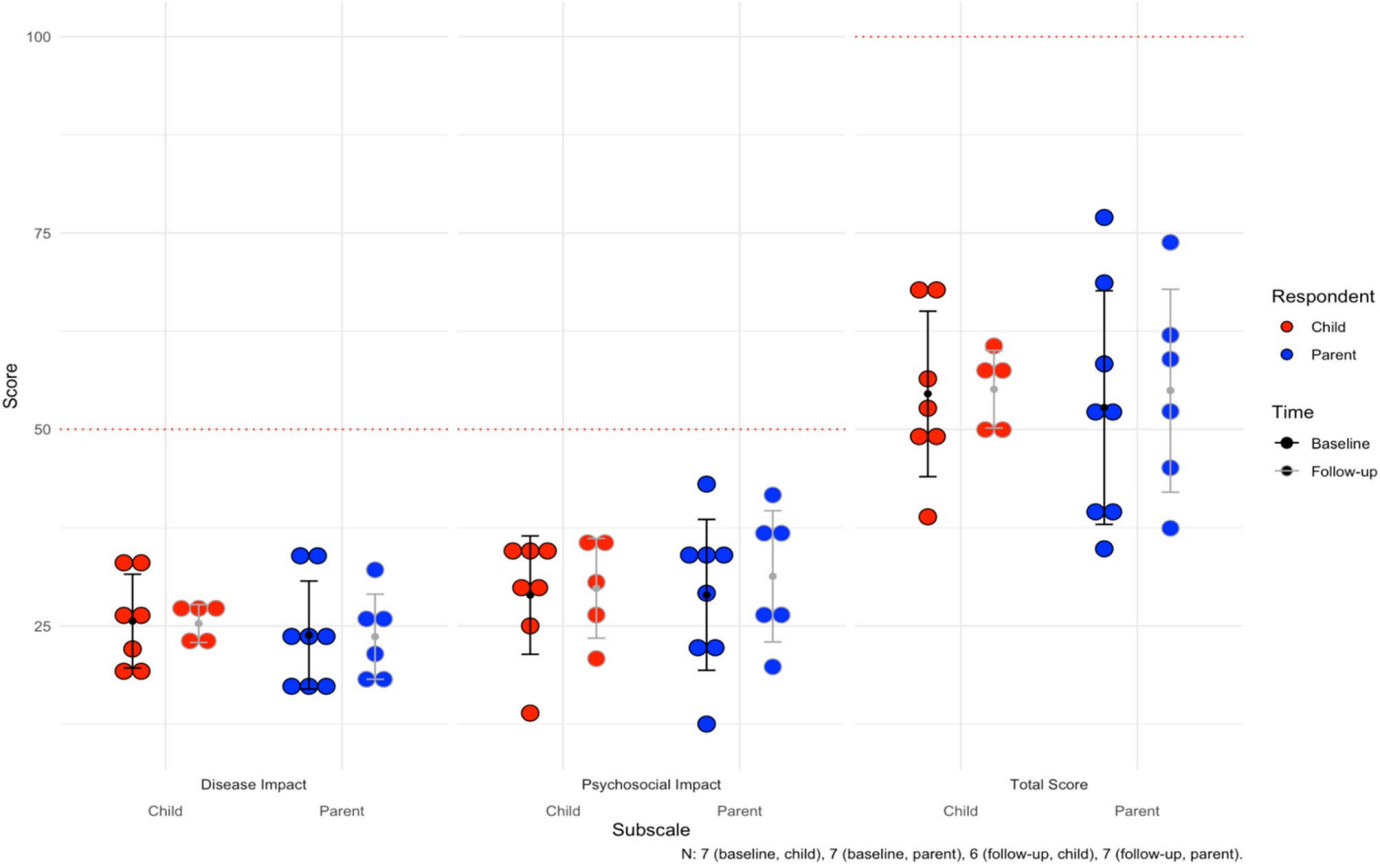
Average subscale and total scores for the Pediatric Cardiac Quality of Life Inventory (PCQLI) across the cohort. For Disease Impact and Psychosocial Impact subscales, 50 is the maximum score (100 for Total Score). The mean follow-up time is 26.6 months. The error bars are the mean and standard deviations for each subgroup

For the parents, there were eight baseline responses (100%, *n* = 8), of which seven had follow-up responses (88%, *n* = 7). Mean follow-up time was 26.6 months. The baseline mean for Disease Impact was 23.8 ± 6.9, with three parents (43%, *n* = 3) reporting lower scores for their children at last follow up. Mean Psychosocial Impact score at baseline was 28.9 ± 9.6, with five parents (71%, *n* = 5) reporting lower scores at last follow-up. Finally, the mean Total Score was 52.8 ± 14.9 at baseline, and four parents (57%, *n* = 4) reported lower total scores for their children at last follow up. The mean scores can be visualized for children and parents in Fig. 1. Notably, average difference between patient and parent Total Scores were 9.1 ± 7.9 and concordance in longitudinal improvements or worsening of scores over time between patients and parents was 75% (*n* = 9).

#### PQLQ

**Fig. 2:**
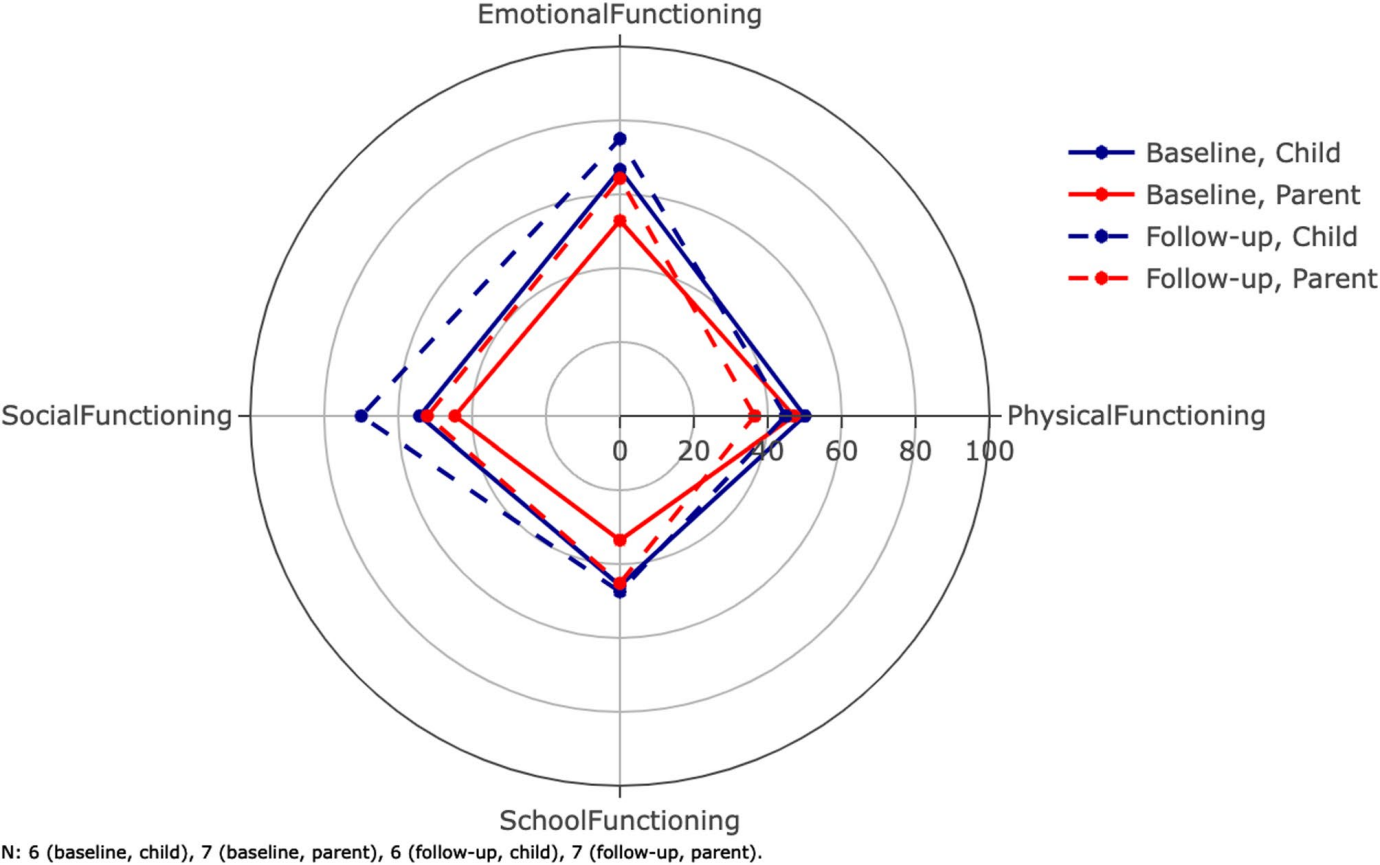
Average scores for the dimensions of the Pediatric Quality of Life Questionnaire (PQLQ). The inventory is filled out by both patients and parents. The parents score their children as functioning worse than the patients personally score themselves. The composite scores total scores are not shown on this radar plot (Table 3). Error bars are not included here in order to maintain visibility. The mean follow-up time was 28.3 months

The average scores for the subscales of the PQLQ can be found in Table 3 (see all responses in Supplementary Table 3). Both patients and their parents responded to this. The max score for all subscales was 100. The mean follow-up time was 28.3 months. Figure 2 shows the scores for physical, emotional, social, and school functioning.

**Table 3:** Average subscale and total scores for the pediatric quality of life questionnaire

Respondent	Child		Parent	
	Baseline (*n* = 7)	Last follow-up (*n* = 5)	Baseline (*n* = 8)	Last follow-up (*n* = 6)
Physical functioning (/100)	*50.0 ± 6.3*	*44.8 ± 6.3*	*47.1 ± 8.4*	*36.5 ± 8.4*
Emotional functioning (/100)	*66.7 ± 24.8*	*75.0 ± 24.8*	*52.9 ± 30.4*	*64.3 ± 30.4*
Social functioning (/100)	*54.2 ± 32.9*	*70.0 ± 32.9*	*44.6 ± 13.7*	*52.1 ± 13.7*
School functioning (/100)	*45.8 ± 30.1*	*47.5 ± 30.1*	*33.6 ± 9.4*	*45.2 ± 9.4*
Psychosocial health summary (/100)	55.3 ± 24.7	64.2 ± 24.7	43.7 ± 16.0	53.9 ± 16.0
Physical health summary (/100)	50.0 ± 6.3	44.8 ± 6.3	47.1 ± 8.4	36.5 ± 8.4
Total (/100)	53.4 ± 17.6	57.4 ± 17.6	44.9 ± 8.6	47.9 ± 8.6

Mean follow-up time was 28.3 months. Scores in *italics* are in Fig. 2

##### Patient responses

Six of eight pediatric patients had baseline responses (75%, *n* = 6), of which five patients had follow-up responses (83%, *n* = 5). For the physical health summary score, which is equal to the physical functioning score, the baseline mean was 50.0 ± 6.3; two patients (40%, *n* = 2) reported lower physical health summary scores at follow-up. For the psychosocial health summary score, the baseline mean was 55.3 ± 24.7; one patient (17%, *n* = 1) reported a lower psychosocial health summary score for themselves at follow-up. For emotional functioning, baseline mean was 66.7 ± 24.8, and two patients (40%, *n* = 2) reported worse functioning at follow-up (Fig. 2). For social functioning, baseline mean was 54.2 ± 32.9, and four patients (60%, *n* = 3) reported worse functioning at follow-up. For school functioning, baseline mean was 45.8 ± 30.1, and four patients (60%, *n* = 3) reported worse functioning at follow-up. Finally, for the total score, baseline mean was 53.4 ± 17.6, and one patient (20%, *n* = 1) reported a lower total score at follow-up.

##### Parent responses

Seven parents had baseline responses (88%, *n* = 7), of which six had follow-up responses (86%, *n* = 6) (Table 3). For the physical health summary score, the mean at baseline was 47.1 ± 8.4. At follow-up, all parents (100%, *n* = 7) reported worse functioning in their child. For the psychosocial health summary score, the baseline mean was 43.7 ± 16.0 and only one parent (17%, *n* = 1) reported a lower follow-up psychosocial health summary score. For emotional functioning, baseline mean was 52.9 ± 30.4, and four parents (67%, *n* = 4) reported worse functioning at follow-up. For social functioning, baseline mean was 44.6 ± 13.7, and four parents (67%, *n* = 4) reported worse functioning at follow-up. For school functioning, baseline mean was 33.6 ± 9.4, and three patients (50%, *n* = 3) reported worse functioning at follow-up. Finally, for the total score, baseline mean was 44.9 ± 8.6, and four parents (67%, *n* = 4) reported lower total scores at follow-up. Average difference between patient and parent total scores was 13.4 ± 9.1. Additionally, concordance in longitudinal changes in patient and parent total scores was 58% (*n* = 5). Parents were more likely to describe physical decline at follow up compared to the patients themselves. Although the remaining emotional, social and school functioning dimensions were more concordant between parents and patients, the total PQLQ parent scores were still lower at follow up compared to patients.

### Cognitive and neuropsychological assessment

In our cohort, all patients (100%, *n* = 9) were affected by neuropsychological symptoms that included: learning difficulties (100%, *n* = 9), attention-deficit hyperactivity disorder (56%, *n* = 5), anxiety (44%, *n* = 4), depression (44%, *n* = 4), autism (22%, *n* = 2) and global developmental delay (11%, *n* = 1).

#### DAS-II

**Fig. 3:**
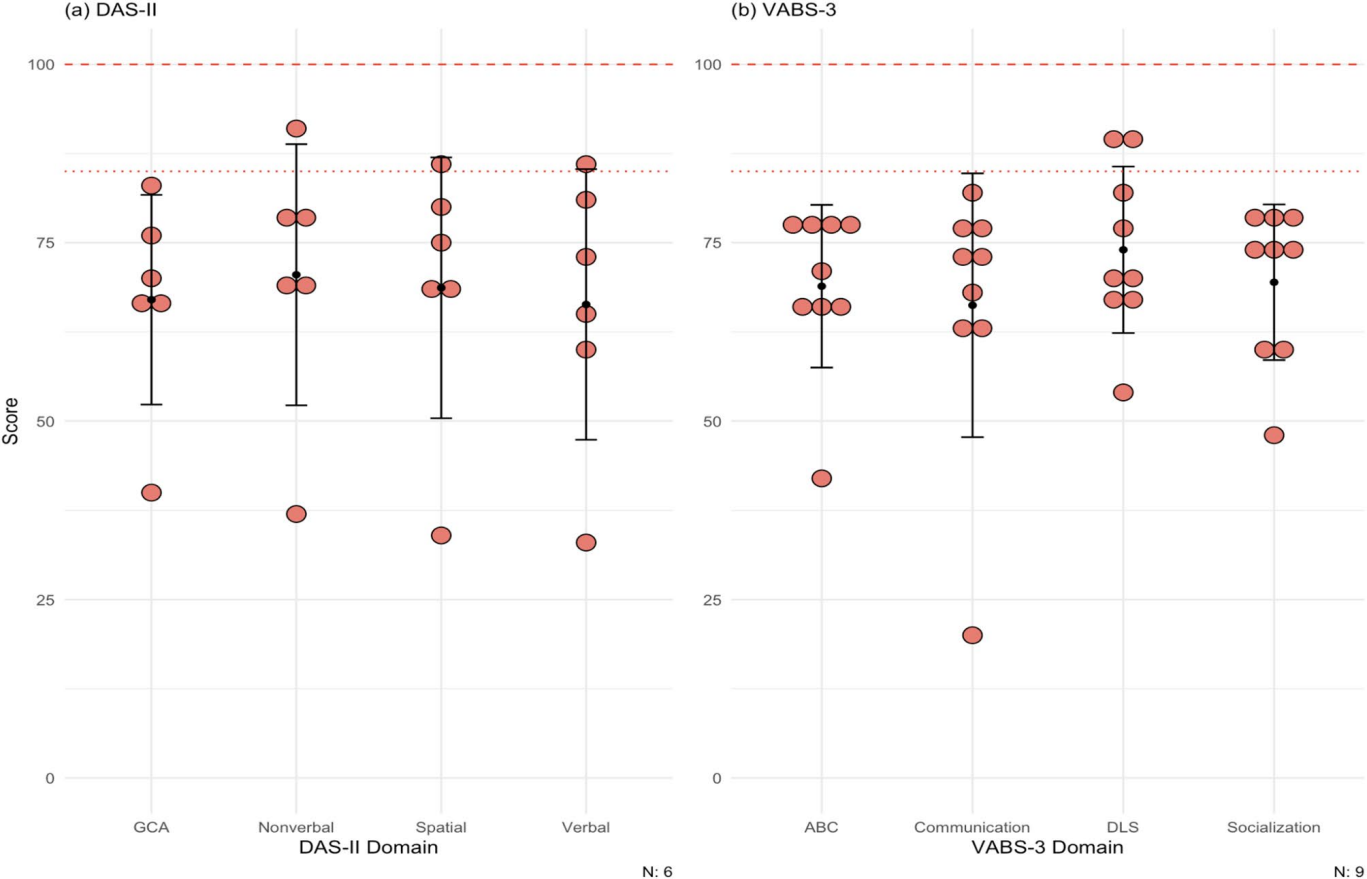
(**a**) Scores for patients for the DAS-II. Population mean and standard deviation for the domains tested are 100 (dashed line) ± 15 (dotted line). Patient 6, 8, 9 did not complete the DAS-II. Most DD patients lie below at least 1 standard deviation on both cognitive tests. (**b**) Scores for patients for the VABS-3. Population means and standard deviations for the domains tested are 100 (dashed line) ± 15 (dotted line). All patients completed this test at baseline only. The maximum score of the test is 170. Most of the patients scored below at least 1 standard deviation on all domains. DAS-II = Differential Ability Scales Second Edition, VABS-3 = Vineland Adaptive Behavior Scales Third Edition, GCA = General Conceptual Ability, ABC = Adaptive Behavior Composite, DLS = daily living skills

Six patients in the cohort completed the DAS-II at baseline. Figure 3a shows the average score for our cohort in each of the domains (see the scores for each domain for each patient in Supplementary Table 4). The mean at baseline for each domain in our cohort was: GCA, 67.0 ± 14.7; verbal, 66.3 ± 19.0; nonverbal, 70.5 ± 18.3; spatial, 68.7 ± 18.3.

#### VABS-3

All patients completed the VABS-3 at baseline. The mean at baseline for each domain in our cohort was: ABC, 68.9 ± 11.4; communication, 66.2 ± 18.5; daily living skills 74.0 ± 11.7; socialization, 69.4 ± 10.9 (Fig. 3b; see the scores for each domain for each patient in Supplementary Table 5).

### Ophthalmological assessment

**Fig. 4:**
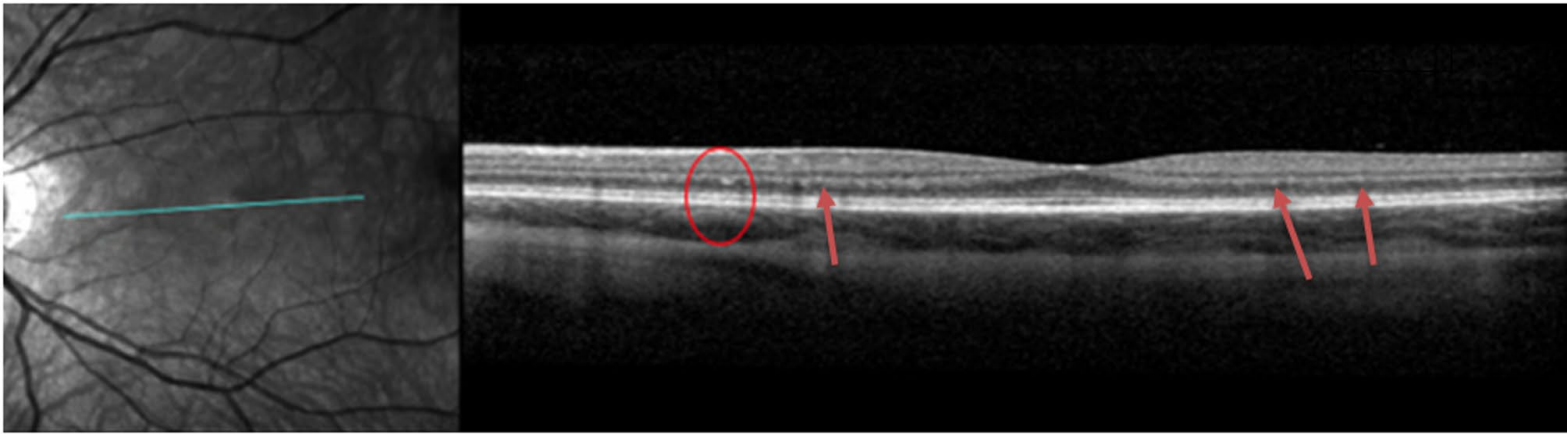
Spectral Domain Optical Coherence Tomography (SD-OCT) of the left eye of patient 5 showing hyper-reflective foci on the border of the outer nuclear layer (ONL) foci and outer plexiform layer (OPL) (red circle and red arrows)

DD patients very commonly have visual disturbances. In our cohort, eight patients had one or more ophthalmological changes (89%, *n* = 8): nyctalopia (11%, *n* = 1), photophobia (11%, *n* = 1), blurry vision (33%, *n* = 3), myopia (78%, *n* = 7), prescribed corrective lenses (78%, *n* = 7), and photophobia (89%, *n* = 8). The BCVA was within the normal range for all patients (100%, *n* = 9). Of note, hyperreflective foci were identified at the level of the outer nuclear layer (ONL) and the border of the outer plexiform layer in six patients (67%, *n* = 6) in SD-OCT (example in Fig. 4). These patients also had paler fundus pseudocolor images with what appeared to be a loss of retinal pigmentation. Fundus autofluorescence imaging demonstrated increased macular autofluorescence.

### Cardiopulmonary assessment

Most patients in the cohort reported cardiac related symptoms (78%, *n* = 7): dyspnea (33%, *n* = 3), palpitations (44%, *n* = 4), and chest pain (56%, *n* = 5). All nine patients had baseline echocardiograms (100%, *n* = 9) and seven patients had follow-up echocardiograms (78%, *n* = 7). Regarding outcomes, one patient underwent transplantation during the study period at age 14. There were no deaths or ventricular assist device implantations. Two pediatric patients had baseline data only; one patient underwent cardiac transplantation and another patient stopped following up due to the COVID pandemic. Due to standardized z-score reporting in pediatric patients, we separated the pediatric patients (*n* = 8) from the adult patient (*n* = 1). The mean follow-up time was 24.5 months for the pediatric patients. Mean EF at baseline was 68.9 ± 4.8%. After the mean follow-up time, all six pediatric patients with follow-ups had lower EF values (100%, *n* = 6). The mean EF at last follow-up was 64.3 ± 8.5%, with an average paired difference of -5.9% which based on the Wilcoxon signed-rank test, was not statistically significant. The adult patient had a baseline EF of 47% and at last follow-up 41%.

For wall thickness, we utilized a maximum wall thickness (MWT) that measured the largest of the septal or posterior wall. The average MWT at baseline for the pediatric patients was 13.6 ± 7.1 mm (z-score = 6.9 ± 6.2; 75% [*n* = 6] of pediatric patients had a z-score > 2 at baseline). At last follow-up, wall thickness increased in all six pediatric patients (100%, *n* = 6). The mean MWT at last follow-up was 15.6 ± 6.8 mm (z-score = 9.4 ± 7.6), with an average paired difference of 3.8 mm, which based on the Wilcoxon signed-rank test, was not statistically signfiicant. For the young adult, his MWT was 30.4 mm at baseline and 28.2 mm at last follow-up after six months.

Mean LVEDD at baseline for the pediatric patients was 37.8 ± 5.7 mm (z-score = -1.6 ± 1.8). At last follow-up, four patients had a smaller LVEDD (67%, *n* = 4). Both patients with increased LVEDD, dilated in isolation of ventricular wall thinning, but did experience a drop in LVEF. The mean at last follow-up for the pediatric patients was 36.5 ± 6.7 mm (z-score = -2.1 ± 1.5), with an average paired difference of -0.5 mm. For the young adult, who had LV wall thinning and a drop in his LVEF during follow up, his LVEDD was 43.1 mm at baseline and 46.2 mm at last follow-up after six months.

**Fig. 5:**
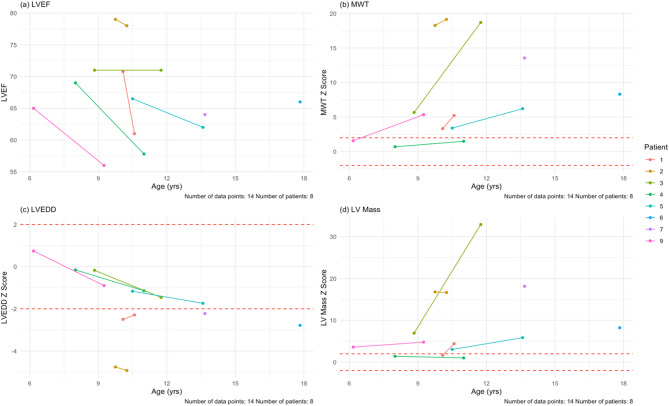
Echocardiogram findings for the pediatric patients in the cohort at their baseline and last follow-up visits. (**a**) LVEF plotted against age in our cohort. There is a downward trend (blue line) as age increases. (**b**) MWT z-scores plotted against age in our cohort. DD hearts thicken over time, as in this cohort. (**c**) LVEDD z-scores plotted against age. There is a downward trend in this age range. (**d**) LV mass z-scores plotted against age in our cohort. Despite the hearts having more mass, the scores remain relatively stable over time. The mean follow-up time for echocardiogram assessment was 24.5 months. MWT = maximum wall thickness, LVEDD = left ventricular end-diastolic dimension

Mean LV mass at baseline for the pediatric patients was 192.7 ± 133.1 g (z-score = 7.5 ± 6.6). Of the pediatric patients in the cohort (*n* = 8), 75% (*n* = 6) had a z-score >2 at baseline. At follow-up (*n* = 6), six patients had increased LV mass (100%, *n* = 6). Mean LV mass at last follow-up was 200.7 ± 88.9 g (z-score = 10.9 ± 12.0), with an average paired difference of + 66.2 g, which based on the Wilcoxon signed-rank test, was not statistically significant. For the young adult, he had an LV mass of 835.1 g at baseline and 789.1 g at follow-up after six months. Mean GLS at baseline for the pediatric patients was − 14.1% ± 2.9% which is lower than the − 20% measured in children without any cardiomyopathy [20]. Four pediatric patients (50%, *n* = 4) had follow-up GLS values, of which two had a GLS that was less negative (50%, *n* = 2). Mean GLS at last follow-up was − 14.7% ± 5.1%, with an average paired difference of + 0.14%. For the adult patient, the GLS at baseline was − 5.8% and at last follow-up was − 3.0%. Of note, this patient’s LVEF was already less than 50% base baseline. All echocardiogram data can be seen in Table 4; Fig. 5, and Supplementary Fig. 1.

**Table 4:** Summary table for cardiac measurements at baseline and at last follow-up, with a mean follow-up time of 24.5 months

Parameter	At baseline (*n* = 8)	At last follow up (*n* = 6)	Average paired difference
Mean age, years	10.6 ± 3.6	11.1 ± 1.5	
Mean height, cm	146.2 ± 25.4	148.3 ± 22.2	+ 11.0
Mean weight, kg	49.5 ± 23.4	48.9 ± 21.1	+ 7.5
NYHA I/II/III	3/5/1	3/5/1	
LVEDD, mm	37.9 ± 5.7	36.5 ± 6.7	-0.5
LVEDD z-score	-1.6 ± 1.8	-2.1 ± 1.5	
GLS, %	-14.1 ± 2.9	-15.2 ± 4.2	+ 0.14
MWT, mm	14.1 ± 6.7	15.6 ± 6.8	+ 3.4
MWT z-score	7.0 ± 6.0	9.4 ± 7.6	
LV EF, %	68.9 ± 4.8	64.3 ± 8.5	-5.9
LV mass, g	194.4 ± 131.1	200.7 ± 88.9	+ 64
LV mass z-score	8.0 ± 6.2	11.0 ± 12.0	

This is for the pediatric patients only (*n* = 8). None of the differences were statistically significant. The critical value for the Wilcoxin signed-rank test is 12 for an n_1_ = 9 and n_2_ = 7. NYHA = New York Heart Association Heart Failure Class, LVEDD = left ventricular end-diastolic dimension, LVESD = left ventricular end-systolic dimension, FS = fractional shortening, GLS = global longitudinal shortening, MWT = maximum wall thickness, EF = ejection fraction

#### Electrocardiogram findings

DD causes dysfunctional macroautophagy that results in accumulation of intracellular vacuoles, a mismatch between supply and demand of energy within cells and eventual cell death. The cardiac hypertrophy and fibrosis that occur result in significant electrophysiologic abnormalities including high voltage with repolarization abnormalities, conduction disorders, accessory pathways, and atrial and ventricular arrhythmias. Therefore, electrocardiogram (ECG) findings at baseline were also assessed in all nine patients. Baseline ECG findings included: Wolff-Parkinson-White (WPW) (56%, *n* = 5), LV hypertrophy (44%, *n* = 4), biventricular hypertrophy (22%, *n* = 1), sinus bradycardia (11%, *n* = 1), and 1st degree atrioventricular (AV) block (11%, *n* = 1).

##### Pulmonary function testing

Most of our patients (89%, *n* = 8) had respiratory related problems: wheezing (44%, *n* = 4), dyspnea (33%, *n* = 3), cough (22%, *n* = 2), asthma allergies (11%, *n* = 1), and sleep apnea (11%, *n* = 1). In our cohort, seven patients underwent PFTs at baseline (78%, *n* = 7) and five patients completed a follow-up visit (71%, *n* = 5). At baseline, the average percent of predicted seated upright FVC was 74.6% ± 8.5% (2.3 ± 1.3 L) (Table 5). After a mean follow-up time of 5.8 months, one patient had a lower upright FVC (20%, *n* = 1), one had no change (20%, *n* = 1), and three had an increase (60%, *n* = 3). The mean percent of predicted upright FVC at last follow-up was 74.0% ± 9.6% (2.4 ± 1.6 L).

**Table 5:** Pulmonary function testing results for the cohort

Parameter	Mean, baseline (*n* = 7)	Mean, follow-up (*n* = 5)	Average paired difference
FVC, upright	2.6 ± 1.3	2.7 ± 1.5	+ 0.2
% of Predicted FVC, upright	74.6 ± 4.5	74.0 ± 9.6	+ 2.8
FVC, supine	2.1 ± 1.1	2.3 ± 1.2	+ 0.3
% of Predicted FVC, supine	60.1 ± 12.6	64.4 ± 14.0	+ 8.2
FEV1	2.3 ± 1.1	2.4 ± 1.2	+ 0.1
% of Predicted FEV1	69.7 ± 14.4	74.4 ± 10.0	+ 8.0
FEV1/FVC	0.89 ± 0.05	0.90 ± 0.07	+ 0.01
% of Predicted FEV1/FVC	104.6 ± 6.2	105.6 ± 7.4	+ 8.0

The mean follow-up time was 5.8 months. FVC = forced vital capacity, FEV = forced expiratory volume

Mean percent of predicted supine FVC at baseline was 60.7% ± 12.6% (1.8 ± 1.2 L). After the mean follow-up time, from the five patients who completed a follow-up visit, one patient had a lower FVC (20%, *n* = 1), one had no change (20%, *n* = 1), and the rest had an increase (60%, *n* = 3). Last follow-up percent of predicted supine FVC was 64.4% ± 14.0% (2.0 ± 1.3 L).

The mean percent of predicted FEV1 was 53.6% ± 16.6% (1.6 ± 1.0 L). At follow-up, two patients had a lower percent of predicted FEV1 (40%, *n* = 2). The mean percent of predicted FEV1 at follow-up was 61.6% ± 16.8% (1.8 ± 1.3 L).

For the FEV1/FVC ratio, the mean percent of predicted was 103.4% ± 1.6% (0.89 ± 0.09). At follow-up, one patient had a lower percent of predicted FEV1/FVC (20%, *n* = 1), one had no change (20%, *n* = 1), and three had an increased (60%, *n* = 3). The mean at follow-up was 108.2% ± 7.7% (0.92 ± 0.05 L).

#### CPET

**Fig. 6:**
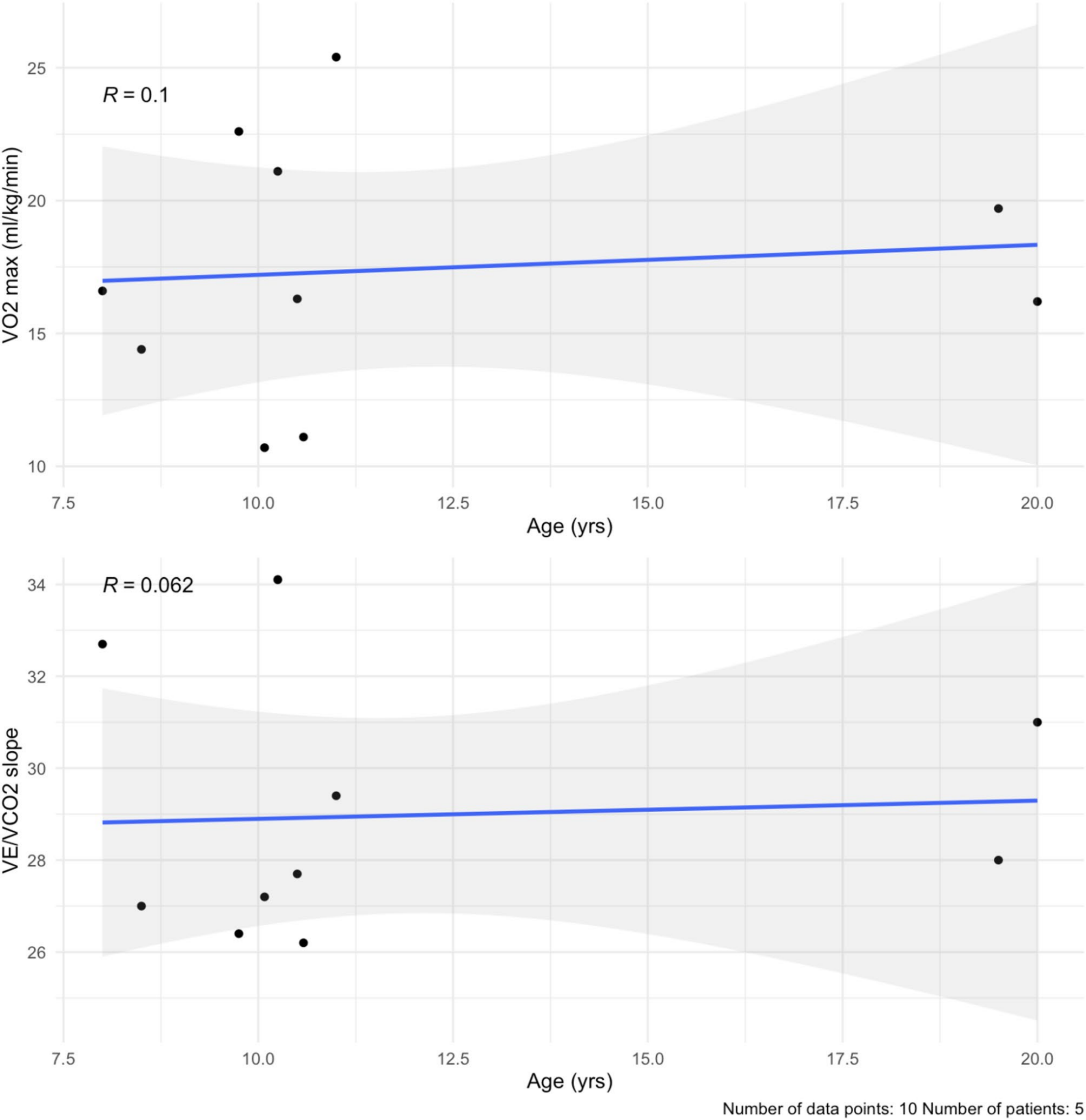
(**a**) VO_2_ max by age. There is a small positive correlation with age (blue line), likely due to lung maturity during puberty. (**b**) V_E_/VCO_2_ slope values plotted over time in the cohort. They remain stable over the 6 months. The mean follow-up time was 5.2 months

In this study, CPET was performed at baseline and at one follow-up visit, with a mean follow-up time of 5.2 months. Five patients performed CPET in our cohort (56%, *n* = 5) at baseline and at one follow-up, for a total of 10 visits. Our cohort had a baseline mean VO_2_ max of 17.2 ± 4.4 ml/kg/min (percent of predicted of 39.0 ± 9.0%) (Table 6; Fig. 6a, Supplementary Table 6). After a mean follow-up time of 5.2 months, three patients had a lower VO_2_ max (60%, *n* = 3). At last follow-up, the mean VO_2_ max was 17.6 ± 5.6 ml/kg/min (percent of predicted of 42.6 ± 13.0%). Out of the 10 total visits, six assessments reached an RQ of ≥ 1.0 (60%, *n* = 6). The baseline mean for ventilatory efficiency (V_E_/VCO_2_ slope) was 28.4 ± 2.5 (Table 6; Fig. 6b, Supplementary Table 6). After the mean follow-up time, two patients had a smaller V_E_/VCO_2_ slope (40%, *n* = 2). At last follow-up, the mean V_E_/VCO_2_ slope was 29.5 ± 3.2.

**Table 6:** Summary of CPET parameters and results of the 6MWT

Parameter	Normal for males	Baseline (*n* = 5)	6-Month follow up (*n* = 5)	Average paired difference
Age at CPET, years	11.2 ± 2.6 [37]	11.6 ± 4.5	12.1 ± 4.5	+ 0.5
Exercise Time, min	-	8.1 ± 4.4	10.4 ± 5.4	+ 2.3
Peak VO_2_ max, mL/kg/min	42.95–49.55 [36] 43.5 ± 9.3 [37]	17.2 ± 4.4	17.6 ± 5.7	+ 0.5
% of Predicted VO2 max	-	39.0 ± 9.0	42.6 ± 13.1	+ 3.6
V_E_/VCO_2_ ratio (AT)	30.1 ± 4.2 [37]	28.4 ± 2.5	29.5 ± 3.2	+ 1.1
Peak RQ	1.14 ± 0.10 [37]	0.98 ± 0.12	1.0 ± 0.01	+ 0.04
6MWT, m	626 ± 65 [39] 691.0 ± 66.3 [40]	448.70 ± 64.70**	405.9 ± 136.1^^	-25.9

None of the differences were statistically significant. Mean follow up time was 5.2 months for the CPET; 23.2 months for the 6MWT. On average, patients walked a shorter distance over the 6 min after follow-up time. 6MWT = 6-minute walk test, RQ = respiratory quotient, AT = anaerobic threshold. **: *n* = 9, ^^: *n* = 7

### Neuromuscular assessment

Seven patients (78%) in our cohort reported neuromuscular symptoms at baseline: delay in motor milestones (67%, *n* = 6), difficulty running (67%, *n* = 6), difficulty walking (67%, *n* = 6), fatigue (67%, *n* = 6), weakness (67%, *n* = 6), joint pain (33%, *n* = 3), difficulty sitting (11%, *n* = 1), hypotonia (11%, *n* = 1), loss of range of motion (11%, *n* = 1), and abnormal gait (11%, *n* = 1). All limbs were found to be affected across the cohort: lower proximal limb (78%, *n* = 7), lower distal limb (67%, *n* = 6), upper proximal limb (56%, *n* = 5), upper distal limb (44%, *n* = 4).

**Fig. 7:**
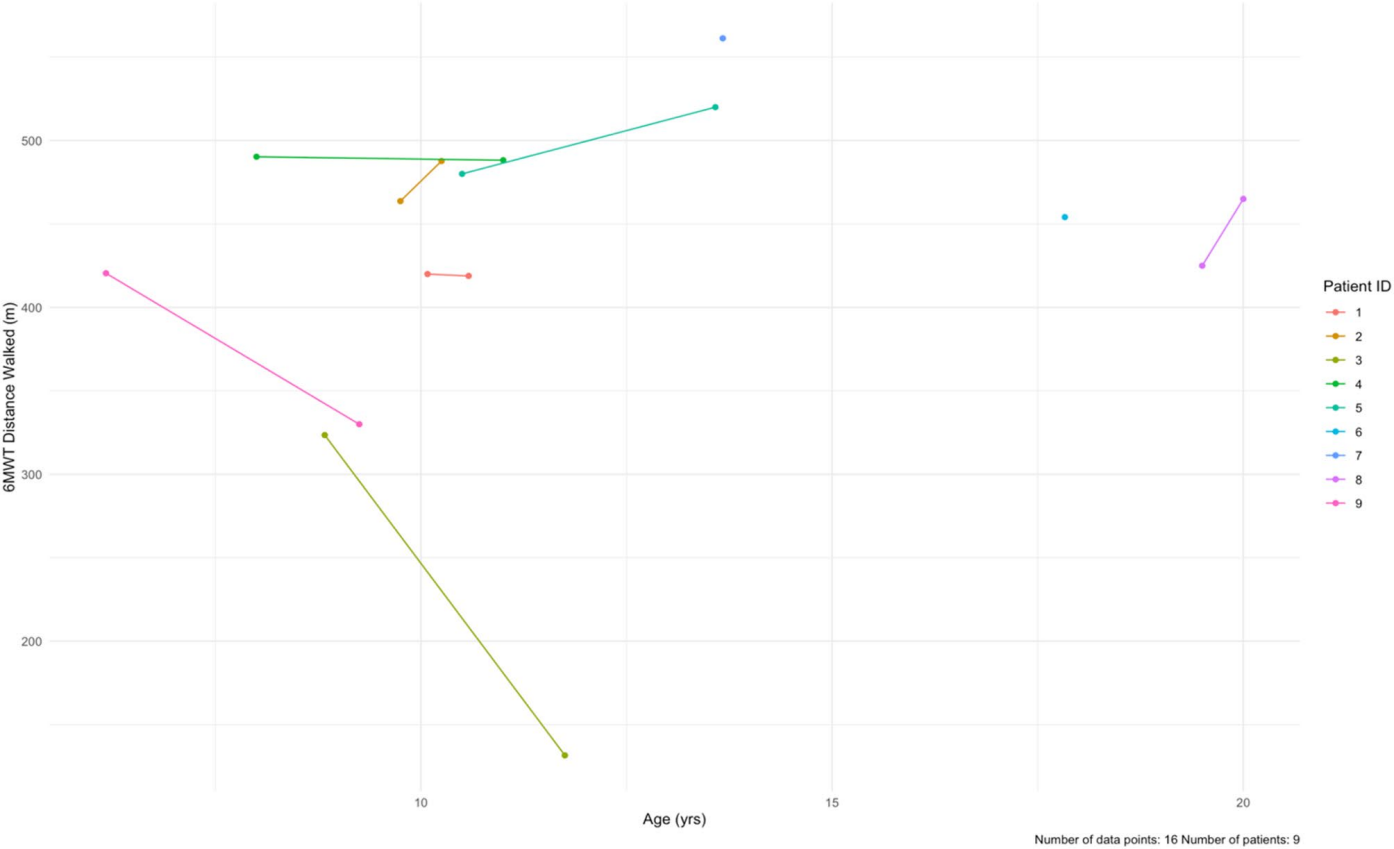
Longitudinal data for the 6-minute walk test (6MWT) at baseline and at follow-up for each patient in the cohort. The data is distance walked (meters) by age. Patients 6 and 7 only had baseline data. The mean follow-up time was 23.3 months

For the 6MWT, all patients had baseline testing (100%, *n* = 9), but only seven patients had follow-up testing (78%, *n* = 7). At baseline, our cohort averaged 448.7 ± 64.7 m (Table 6; Fig. 7, Supplementary Table 7). After a mean follow-up time of 23.3 months, four patients walked shorter distances (57%, *n* = 4). The mean distance at last follow-up was 405.9 ± 136.1 m, with an average paired difference of -25.9 m.

**Fig. 8:**
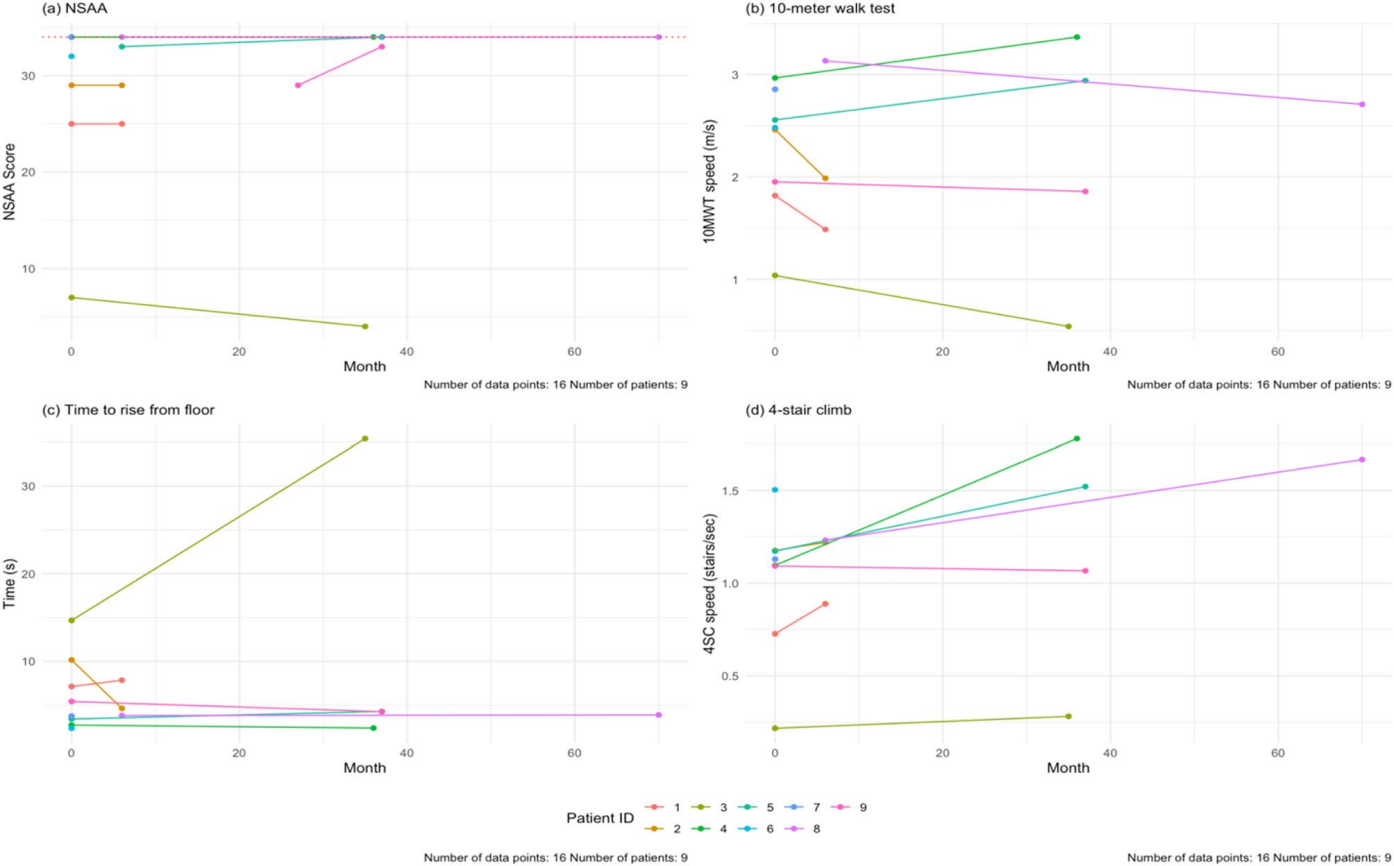
(**a**) Baseline and last follow-up NSAA scores for the patients in the cohort. The maximum score is 34 (dashed line). The NSAA trended up with age (blue line). (**b**) Baseline and last follow-up speed during the 10MWT for our cohort. There is a positive correlation with age. (**c**) Baseline and last follow-up speed during the 4SC in our cohort. There is a positive correlation with age. The mean follow-up time for all these tests is 23.1 months. NSAA = North Star Ambulatory Assessment, 10MWT = 10-meter walk test, 4SC = 4-stair climb test

The results for the NSAA, 10MWT, time to rise from floor, and 4SC tests are shown in Fig. 8. For the NSAA, all patients completed the test at baseline (100%, *n* = 9). Seven patients completed follow-up visits (78%, *n* = 7), with a mean follow-up time of 23.1 months. The average NSAA total score at baseline was 28 ± 9 out of a max total of 34. After the follow-up time, one patient had a worse NSAA total score (14%, *n* = 1) and two patients had better scores (29%, *n* = 2) (Supplementary Table 8, Supplementary Fig. 4a). Out of the seven patients who had follow-up data, four (57%, *n* = 4) had no changes over 6-, 12-, 24-, and 36-month follow-ups. One of our patients, Patient 3, had markedly worse NSAA total score and 10MWT and 4SC times. Notably this patient had difficulty following instructions, which may have impacted these scores (Supplementary Fig. 4).

For the 10MWT, all nine patients (100%, *n* = 9) completed the test at baseline, but only seven (78%, *n* = 7) patients had follow-up testing done. The mean follow-up time was 19.4 months. In our cohort, ages 6–20, the baseline average speed was at 2.4 ± 0.6 m/s. At the end of study, two patients were slower (29%, *n* = 2). The average speed at follow-up was 2.2 ± 1.0 m/s.

For the time to rise from floor test, nine patients completed the test at baseline (100%, *n* = 9) and seven patients completed follow-up testing (78%, *n* = 7). The mean follow-up time was 23.3 months. The average time taken at baseline was 5.9 ± 4.1 s. At the end of the study, three patients took longer to rise from the floor (43%, *n* = 3). The mean time at last follow-up was 8.9 ± 11.8 s.

For the 4SC, we had nine patients complete the test at baseline (100%, *n* = 9), and seven patients with follow-up visits (78%, *n* = 7). The average speed at baseline was 1.1 ± 0.4 stairs/sec. After the mean follow-up time of 19.4 months, two patients were slower (29%, *n* = 2) (Fig. 8d).

### Laboratory parameters

**Fig. 9:**
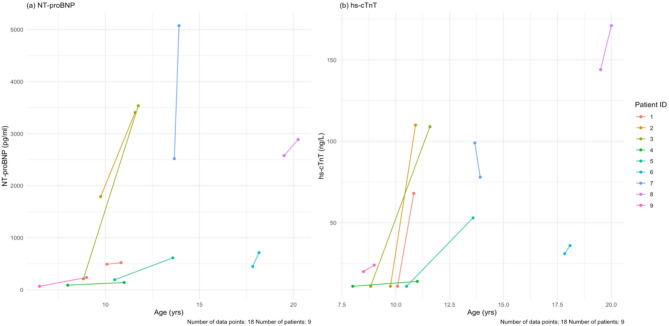
(**a**) Baseline and last follow-up NT-proBNP values for each patient. (**b**) Baseline and last follow-up hs-cTnT values for each patient. The mean follow-up time for all these tests is 18.7 months. NT-proBNP = N-terminal pro brain natriuretic peptide, hs-cTnT = high sensitivity cardiac troponin T

All patients completed the baseline laboratory measurements and follow-up with a mean follow-up time of 18.6 months. Of note, cardiac biomarkers including N-terminal pro brain natriuretic peptide (NT-proBNP) and high sensitivity cardiac troponin T (hs-cTnT) were elevated at both baseline and follow-up. Similarly, skeletal biomarkers including CPK and aldolase were similarly elevated at baseline and follow-up, as were AST, ALT and LDH (Table 7). Figure 9 shows the values for NT-proBNP and hs-cTnT for all patients at baseline and follow-up only, where mean paired difference was 971.3 pg/mL and 37.3 respectively, which based on Wilcoxon signed-rank test was a significant increase in NT-proBNP, but not hs-cTnT; Supplementary Fig. 5 shows the values at all visits.

**Table 7:** Lab values for the cohort, summarized at baseline and at last follow-up

Parameter	Mean, baseline (*n* = 9)	Mean, follow-up (*n* = 9)	Average paired difference	Normal range
NT-proBNP (pg/mL)	932.2 ± 1055.9	1903.6 ± 1831.2	+ 971.3	< 125
CPK (U/L)	1393.8 ± 831.0	1340.9 ± 794.5	+ 62.7	39–308
AST (U/L)	407.0 ± 175.2	361.9 ± 177.2	-45.1	0–40
ALT (U/L)	259.7 ± 110.6	301.3 ± 134.3	+ 41.7	0–41
Creatinine (mg/dL)	0.6 ± 0.2	0.7 ± 0.3	+ 0.1	0.67–1.17
Aldolase (units/L)	21.9 ± 7.7	21.4 ± 9.0	-0.5	1.0–7.5
LDH (U/L)	1069.3 ± 346.1	1223.3 ± 565.0	+ 154.0	140–280
hs-cTnT (ng/L)	41.1 ± 51.5	78.4 ± 51.2	+ 37.3	< 14

Individual patient labs can be found in the appendix. A summary of the baseline lab values for the cohort, with a mean follow up time of 18.7 months. Figure 9 shows the baseline and follow-up values for all patients for NT-proBNP and hs-cTnT. Supplementary Fig. 5 shows values for all visits that were recorded. NT-proBNP = N-terminal pro brain natriuretic peptide, CPK = creatine phosphokinase, AST = aspartate transaminase, ALT = alanine transaminase, LDH = lactate dehydrogenase, hs-cTnT = high sensitivity cardiac troponin T

## Discussion

Overall findings from this deep phenotyping study confirm that DD is a multisystem disease with significant morbidity across multiple organs. Notably baseline abnormalities and deficits were present in this predominantly pediatric population and continued to progress through the course of the study. Correlating with this, patient reported outcomes scores were lower than those reported in healthy children as well as children with other cardiac diseases. Though only limited follow up was available, this study still suggests variability in rates of disease progression in the various organs as well as disease trajectory between patients. Findings will now be discussed on a system-by-system basis below.

### Patient reported outcome measures

Of the patient reported outcomes instruments used in this study, only the PCQLI has been validated in patients with cardiac disease [4]; the PQLQ has been validated in pediatric cancer patients [11, 21]. Both instruments assess patient perceptions on disease perspectives and also physical and psychosocial domains. The most notable findings from this study were the low scores in both instruments by both patients and parents, where average total PCQLI and PQLQ scores at baseline and follow up were < 60 for both patients and parents. To provide a context for comparison, in the PCQLI instrument, mean total score ranged between 60 and 80 in patients with congenital heart disease not requiring catheter or surgical correction, while in the PQLQ instrument, self-identified healthy children reported a mean score of 83.9 ± 12.5 (*n* = 5079, ages: 8 to 16) [21] and children with cancer on treatment a mean score of 68.9 ± 16 [22]. These studies provide some insight into the quality of life that Danon patients and their parents’ report. The overall scores reported by DD patients are substantially lower than those reported by children with serious and/or chronic illnesses. Further studies are needed to validate these instruments in DD patients and other multisystemic diseases that affect neurocognitive, musculoskeletal and cardiac functioning.

#### Cognitive assessment

Intellectual disability is a hallmark of affected male DD patients, with more than 70% experiencing a degree of cognitive impairment [23, 24]. Only one study to date has delved into the cognitive and neuropsychological aspects of patients with DD [24], where the authors used the Cognitive Neuropsychiatric Battery and Wechsler Intelligence test. In regard to neuropsychological disorders, mood disorders such as depression and anxiety have been reported in DD patients [24]. Population mean scores for both the DAS-II [25, 26] and VABS-3 [27] is 100 ± 15. DD patients in this cohort scored on average two standard deviations below the mean on cognitive and behavioral standardized testing, suggesting significant impairment. For comparison, patients with Fragile X syndrome had a mean VABS-3 of 50.8 ± 20.6 and patients with 22q11.2 deletion syndrome had a mean of 64.3 ± 17.1 [17].

#### Ophthalmological assessment

DD patients often have structural and functional ophthalmic deficits; however, this presentation is variable. Retinal pathology in DD patients increases with age, and males have more abnormalities compared to affected females [28]. A previous study suggested that pigmentary retinopathy could indicate the presence of pathogenic *LAMP2* variants, even without other clinical findings [28]. Most of our patients had loss of retinal pigmentation as evidenced by increased macular autofluorescence, a sign of retinal pigment epithelium dysfunction. Furthermore, hyperreflective foci, which indicate retinal disease, on the border of the ONL and outer plexiform layer, as seen in Fig. 4, is another ophthalmological abnormality seen previously on SD-OCT in DD patients and also seen in our cases [28]. Given the severity of these symptoms, we believe there is a pressing unmet need for ophthalmologic surveillance and therapies for DD.

#### Cardiac assessment

Cardiac assessments of our cohort of DD patients revealed declining EF, thickening myocardium, decreasing LVEDDs, and increasing LV mass over time. DD patients have been shown to have increased wall thickness that sometimes progresses to dilated cardiomyopathy [29]. Our cohort was comprised of eight pediatric patients (mean follow-up time of 24.5 months) and one adult patient (follow-up time of six months). One pediatric patient was transplanted who only had baseline data: a normal EF of 64%, LVH with a MWT z-score of 13.6, LVEDD z-score of -2.2, LV mass z-score of 18.1 with a GLS of -9.5%.

Most notably, all patients in the cohort who had follow-up echocardiograms had a decrease in LVEF over time, which is consistent with what has been previously reported in DD [30, 31]. Of the seven pediatric non-transplanted patients, based on z-scores six patients had left ventricular hypertrophy and elevated LV mass at baseline. Of the pediatric patients with follow-up, all had progressive hypertrophy except for one patient and half had increases in their LV mass. For LVEDD, only one patient of the six pediatric patients had progressive dilation. Given the significant progressive changes in LVEF, LV wall thickness and LV mass, these parameters should be considered as possible surrogate markers for disease progression.

##### Pulmonary function tests

As a syndrome characterized by cardiac and skeletal muscle involvement, DD may impact respiratory function due to both cardiac and respiratory muscle impairments. PFTs, specifically a decline in FVC percent predicted over time is a well-documented and validated outcome measure in Pompe disease, a glycogen storage disorder that results in a myopathy as well [32, 33]. In our DD cohort, FVC percent of predicted, FEV1, and FEV1/FVC ratio were within normal range at baseline in our group with a majority having improvements in these parameters over time, suggesting that significant myopathy affecting respiratory function was not present.

##### CPET

The CPET evaluates functional capacity, and is used as a prognostic tool in cardiomyopathy patients [34, 35]. Our cohort had a baseline mean of 17.2 ± 4.4 ml/kg/min, which was lower than that what has been reported in healthy kids. In a study of healthy children, the peak VO2 range was 43.0–49.6 ml/kg/min for boys aged 10–14 years old, with a cut-off of < 38.7 ml/kg/min being used to identify individuals with weaker cardiorespiratory fitness [36]. These mean values aligned with another pediatric study [37]. In both studies, the respiratory exchange ratio (RER), or respiratory quotient (RQ), was ensured to be >1.0. In our study RQ >1.0 was met in 60% of the CPETs completed. Thus, while mean peak oxygen consumption in our cohort of male patients is poor, interpretation is limited due to variability in mean respiratory quotient between and within patients. Furthermore, exercise tolerance and measured exertional capacity can be confounded by the neuromuscular manifestations DD patients can have, thus V_E_/VCO_2_ slope, which is not dependent on RER, may be a better measure of cardiopulmonary mechanics than peak VO2. Notably, VE/VCO2 slope in this cohort was comparable to the 29.3 ± 4.8 that was found in healthy pediatric controls in a different study [38]. It is worth noting that out of 10 total CPETs conducted, only 2 were terminated due to leg weakness (reached RQ >1.0), whereas the others were for shortness of breath or fatigue.

### Neuromuscular assessment

Neuromuscular assessment in this study was completed via 6MWT and NSAA which includes 10MWT and 4SC testing. Notably, given the expected musculoskeletal involvement in these patients, testing in this cohort of DD patients was consistent with functional limitations and proximal muscles weakness. Specifically, baseline mean 6MWT distance was 448.7 ± 64.7 m and decreased by nearly a third at follow up. As a reference, several studies of healthy adolescents aged < 18 years old mean 6MWT was upwards of 600 m [39, 40]. In a study in the Netherlands that looked at 6MWT in children with dilated cardiomyopathy, but no neuromuscular involvement, the 6MWT was 448 ± 144 m [41], which is comparable to the averages in our cohort. The 6MWT has been shown to be correlated (*r* = 0.4) with VO_2_ max [39] and thus can be complementary test to CPET if RER is not achieved. Importantly though, the 6MWT results can also be confounded by neuromuscular symptoms.

The NSAA total score and 10MWT and 4SC test results for the cohort are shown in Fig. 8. While some of the patients in the cohort reached a score of 34 on the NSAA (Fig. 8a, Supplementary Fig. 4a), the majority of patients scored below. In a study of Duchenne muscular dystrophy (DMD) patients aged 8.3 ± 3.3 years, those with the slowest progression of the disease had a mean NSAA score of 26.3 ± 5.5 at baseline [42]. Our cohort had mean NSAA scores of 28.6 at baseline and 27.6 at last follow-up, which is comparable to these slowly progressive DMD patients. Both the time to rise from floor and 4SC times in this DD cohort suggested impaired proximal muscle strength. Time to rise from floor has already been utilized to quantify motor function in DMD patients [43] and has been used as a secondary endpoint in DMD drug trials [44]. For 4SC testing, average speeds in healthy males aged 4–16 years were 2.2 stairs/sec − 4.3 stairs/sec [45], with older children being faster. Our cohort’s mean stair ascent rate at baseline was only of 1.1 ± 0.4 stairs/sec, and while the majority of patients improved by last follow up, their rate remained below 2.2 stairs/sec. The NSAA total score, time to rise from floor and 4SC tests are more specific assessment of motor function and should be used to monitor progression of musculoskeletal involvement in these patients. Of interest, 10MWT times in our cohort were comparable to those reported in a study of healthy children aged 6–12 years [46]. These results are consistent with DD preferentially affecting the proximal muscles.

### Laboratory parameters

Cardiac and musculoskeletal biomarkers, including troponin, natriuretic peptides, aldolase, CPK, LDH, and transaminases were all elevated at baseline and follow-up in this DD cohort (Table 7). Although hypertrophy appeared to be accompanied by higher natriuretic peptide and troponin levels, the small sample size and limited number of serial assessments preclude definitive assessment. Additionally, there was substantial inter- and intra-patient variability which led to high standard deviations and skewed means (Table 7, Supplementary Table 9). Thus, these biomarkers may identify episodes of acute disease exacerbations and may be useful for standardizing patients at time of enrollment into comparative effectiveness trials. Additionally, trends in these biomarkers and in particular persistent elevations may identify progressive disease and predict poorer long-term outcomes. Thus, consideration of these biomarkers in clinical trial design, either in eligibility criteria or as surrogates for hard clinical outcomes and evaluating their association with end stage heart failure should be prioritized.

## Limitations

Across rare diseases, small populations and heterogeneity in patient disease expression may impact generalizability of findings in this study to the larger population. Accordingly, a limitation of this study is the small sample size, varied patient ages and disease severity at time of enrollment. The majority of patients in this cohort were pediatric which reflects the severity of this X-linked dominant disease. There were two patients who crossed over into a therapeutic gene therapy trial which necessitated study disenrollment. Altogether, only descriptive analyses could be presented. Furthermore, due to disruption in follow-up because of the COVID-19 pandemic, not all patients returned at regular follow-up intervals, and one patient disenrolled due to safety concerns associated with traveling in the post-COVID-19 era. The absence of standardized follow-up intervals for subjects in a study introduces limitations to comparisons between patients, as well as overall reliability and interpretability. Variability in follow-up intervals made it that data could not be summarized in standardized intervals. By presenting the patient’s baseline parameters as well as last follow-up, we nonetheless derive insights into temporal changes in patients’ characteristics and disease progression.

## Conclusion

Given the scarcity of data and the limited number of individuals affected by Danon disease, natural history studies provide a crucial foundation for understanding this disease’s progression, variability, and clinical manifestations. This study confirms that Danon disease in males manifests with significant progressive cardiac and musculoskeletal symptoms that result in lower patient and parent perceived quality of life. Patient reported outcomes such as the PCQLI and PQLQ need to be further validated in genetic cardiomyopathies involving extracardiac systems. Additionally, to inform treatment decisions as well as clinical trial design, structural and biomarker parameters predicting disease severity and progression should be used to define disease state and risk stratify patients. Natural history studies can enable more informed clinical trial design by clarifying eligibility criteria and identifying surrogate endpoints, but also serve as an important adjunct to therapeutic trials by providing external controls for comparisons.

## Electronic supplementary material

Below is the link to the electronic supplementary material.

Supplementary Material 1

## Data Availability

All data generated or analyzed during this study are included in this published article and its supplementary information files.

## Abbreviations

DD: Danon disease
LV: Left ventricle
LAMP-2: Lysosomal-associated membrane protein 2
LAMP2: LAMP-2 gene
HCM: Hypertrophic cardiomyopathy
PCQLI: Pediatric Cardiac Quality of Life Inventory
PQLQ: Pediatric Quality of Life Questionnaire
DAS-II: Differential Ability Scales, Second Edition
VABS-3: Vineland Adaptive Behavior Scales, Third Edition
NYHA: New York Heart Association Heart Failure Class
LVEDD: Left ventricular end-diastolic dimension
FS: Fractional shortening
GLS: Global longitudinal shortening
MWT: Maximum wall thickness
EF: Ejection fraction
FVC: Forced vital capacity
CPET: Cardiopulmonary exercise testing
6MWT: 6-minute walking distance test
NSAA: North Star Ambulatory Assessment
10MWT: 10-meter walk test
4SC: 4-stair climb test
DMD: Duchenne muscular dystrophy
NT-proBNP: N-terminal pro brain natriuretic peptide
hs-cTnT: High sensitivity cardiac troponin T
