# Supplementary material for: Danon disease in male patients: a prospective natural history study to augment understanding of the phenotype
Source: Orphanet J Rare Dis. 2025 Oct 21;20:530. doi: 10.1186/s13023-025-04058-8 (PMC12542076; doi:10.1186/s13023-025-04058-8)
Supplement: Supplementary file 1 — Supplementary Material 1 [file 13023_2025_4058_MOESM1_ESM.docx]

# Supplementary

## Visit completion

##### Supplementary Table 1. The list of assessments performed in this study, the number of expected visits and number of completed visits pre- and post-COVID pandemic. Pre-COVID encompasses 2/2019-5/2020 and post-COVID encompasses 11/2021-7/2022. *PCQLI = Pediatric Cardiac Quality of Life Inventory, PQLQ = Pediatric Quality of Life Questionnaire, DAS-II = Differential Ability Scales, Second Edition, VABS-3 = Vineland Adaptive Behavior Scales, Third Edition, PFTs = pulmonary function tests, CPET = cardiopulmonary exercise testing, 6MWT = 6-minute walk test.*

| **Assessment** | **Pre-COVID Visits** | | | **Post-COVID Visits** | | |
| --- | --- | --- | --- | --- | --- | --- |
|  | **Expected** | **Completed** | **%** | **Expected** | **Completed** | **%** |
| **Patient reported outcomes (****PCQLI, PQLQ)** | 34 | 24 | 70.5 | 46 | 15 | 34.1 |
| **Cognitive (DAS-II, VABS-3)** | 10 | 9 | 90 | 26 | 0 | 0 |
| **Echocardiogram** | 17 | 14 | 82.4 | 46 | 11 | 23.9 |
| **Pulmonary function tests** | 17 | 14 | 82.4 | 46 | 0 | 0 |
| **CPET** | 17 | 11 | 64.7 | 46 | 1 | 2.2 |
| **6MWT** | 17 | 15 | 88.2 | 46 | 11 | 23.9 |
| **Neuromuscular** | 17 | 14 | 82.4 | 46 | 11 | 23.9 |
| **Laboratory parameters** | 32 | 27 | 84.38 | 85 | 25 | 29.4 |

## Patient reported outcome measures

### PCQLI

##### Supplementary Table 2. Pediatric Cardiac Quality of Life Inventory (PCQLI) scores for patients and their parents at baseline and all attended follow-up visits.

| **Patient ID** | **Age (yrs)** | **Follow-up Month** | **Responder** | **Disease Impact** | **Psychosocial Impact** | **Total Score** |
| --- | --- | --- | --- | --- | --- | --- |
| 1 | 10.64 | 6 | Child | 33.9 | 34.7 | 68.7 |
| 1 | 10.64 | 6 | Parent | 33.9 | 34.7 | 68.7 |
| 1 | 11.2 | 12 | Child | 25.9 | 34.7 | 60.6 |
| 1 | 11.2 | 12 | Parent | 21.4 | 37.5 | 58.9 |
| 2 | 10.28 | 6 | Child | 32.1 | 34.7 | 66.9 |
| 2 | 10.28 | 6 | Parent | 23.2 | 29.2 | 52.4 |
| 2 | 10.93 | 12 | Child | 25.9 | 30.6 | 56.5 |
| 2 | 10.93 | 12 | Parent | 25.9 | 36.1 | 62.0 |
| 3 | 9.34 | 6 | Parent | 25.0 | 33.3 | 58.3 |
| 3 | 9.91 | 12 | Parent | 26.8 | 43.1 | 68.8 |
| 3 | 11.36 | 30 | Parent | 22.3 | 34.7 | 57.0 |
| 3 | 11.86 | 36 | Parent | 18.8 | 26.4 | 45.1 |
| 4 | 8.07 | 0 | Child | 25.0 | 13.9 | 38.9 |
| 4 | 8.07 | 0 | Parent | 22.3 | 12.5 | 34.8 |
| 4 | 8.55 | 6 | Child | 24.1 | 23.6 | 47.7 |
| 4 | 8.55 | 6 | Parent | 19.6 | 19.4 | 39.1 |
| 4 | 9.04 | 12 | Child | 17.0 | 23.6 | 40.6 |
| 4 | 9.04 | 12 | Parent | 24.1 | 18.1 | 42.2 |
| 4 | 10.6 | 30 | Child | 25.0 | 29.2 | 54.2 |
| 4 | 10.6 | 30 | Parent | 27.7 | 25.0 | 52.7 |
| 4 | 11.1 | 36 | Child | 28.6 | 20.8 | 49.4 |
| 4 | 11.1 | 36 | Parent | 25.9 | 26.4 | 52.3 |
| 5 | 10.58 | 0 | Child | 17.9 | 30.6 | 48.4 |
| 5 | 10.58 | 0 | Parent | 17.0 | 23.6 | 40.6 |
| 5 | 11.06 | 6 | Child | 17.0 | 1.4 | 18.4 |
| 5 | 11.06 | 6 | Parent | 17.0 | 11.1 | 28.1 |
| 5 | 11.58 | 12 | Child | 28.6 | 15.3 | 43.9 |
| 5 | 11.58 | 12 | Parent | 20.5 | 12.5 | 33.0 |
| 5 | 13.12 | 30 | Child | 19.1 | 27.1 | 46.2 |
| 5 | 13.12 | 30 | Parent | 14.7 | 25.0 | 39.7 |
| 5 | 13.62 | 36 | Child | 22.1 | 36.5 | 58.5 |
| 5 | 13.62 | 36 | Parent | 17.7 | 19.8 | 37.4 |
| 6 | 17.84 | 0 | Child | 22.1 | 34.4 | 56.4 |
| 6 | 17.84 | 0 | Parent | 17.7 | 20.8 | 38.5 |
| 7 | 13.68 | 0 | Child | 20.6 | 29.2 | 49.8 |
| 7 | 13.68 | 0 | Parent | 17.7 | 34.4 | 52.0 |
| 9 | 8.46 | 24 | Child | 27.7 | 25.0 | 52.7 |
| 9 | 8.46 | 24 | Parent | 33.9 | 43.1 | 77.0 |
| 9 | 8.71 | 30 | Child | 24.1 | 26.4 | 50.5 |
| 9 | 8.71 | 30 | Parent | 32.1 | 41.7 | 73.8 |

### PQLQ

##### Supplementary Table 3. Pediatric Quality of Life Questionnaire (PQLQ) scores for patients and their parents at baseline and all attended follow-up visits.

| Patient Details | | | | Functioning score | | | | Summary score | | Total |
| --- | --- | --- | --- | --- | --- | --- | --- | --- | --- | --- |
| ID | Age | Month | Respondent | Physical | Emotional | Social | School | Psychosocial health | Physical health | Total |
| 1 | 10.2 | 0 | Child | 59.4 | 100 | 85 | 90 | 91.7 | 59.4 | 80.4 |
| 1 | 10.2 | 0 | Parent | 31.3 | 100 | 75 | 50 | 75 | 31.3 | 59.8 |
| 1 | 10.6 | 6 | Child | 34.4 | 100 | 100 | 40 | 80 | 34.4 | 64.1 |
| 1 | 10.6 | 6 | Parent | 31.3 | 95 | 75 | 35 | 68.3 | 31.3 | 55.4 |
| 1 | 11.2 | 12 | Child | 37.5 | 85 | 80 | 70 | 78.3 | 37.5 | 64.1 |
| 1 | 11.2 | 12 | Parent | 28.1 | 90 | 65 | 31.3 | 62.1 | 28.1 | 50.3 |
| 2 | 9.8 | 0 | Child | 46.9 | 65 | 100 | 70 | 76.8 | 46.9 | 65.9 |
| 2 | 9.8 | 0 | Parent | 53.1 | 50 | 40 | 40 | 43.3 | 53.1 | 46. |
| 2 | 10.3 | 6 | Child | 40.6 | 90 | 35 | 25 | 50 | 40.6 | 46.7 |
| 2 | 10.3 | 6 | Parent | 18.8 | 60 | 20 | 20 | 33.3 | 18.8 | 28.3 |
| 2 | 10.9 | 12 | Child | 50.0 | 100 | 80 | 60 | 80 | 50 | 69.6 |
| 2 | 10.9 | 12 | Parent | 21.8 | 40 | 40 | 60 | 46.7 | 21.9 | 38.0 |
| 3 | 8.8 | 0 | Parent | 45 | 75 | 37.5 | 25 | 45.8 | 45 | 45.5 |
| 3 | 9.3 | 6 | Parent | 12.5 | 50 | 30 | 31.3 | 37.1 | 12.5 | 28.5 |
| 3 | 9.9 | 12 | Parent | 10.7 | 56.3 | 40 | 30 | 42.1 | 10.7 | 31.2 |
| 3 | 11.4 | 30 | Parent | 18.8 | 65 | 35 | 55 | 51.7 | 18.8 | 40.2 |
| 3 | 11.9 | 36 | Parent | 15.6 | 75 | 40 | 35 | 50 | 15.6 | 38.0 |
| 4 | 8.1 | 0 | Child | 43.8 | 25 | 10 | 40 | 25 | 43.8 | 31.5 |
| 4 | 8.1 | 0 | Parent | 53.1 | 10 | 35 | 25 | 23.3 | 53.1 | 33.7 |
| 4 | 8.6 | 6 | Child | 25.0 | 40 | 0 | 0 | 13.3 | 25 | 17.4 |
| 4 | 8.6 | 6 | Parent | 50.0 | 45 | 30 | 15 | 30 | 50 | 37.0 |
| 4 | 9.0 | 12 | Child | 56.3 | 55 | 25 | 75 | 51.7 | 56.3 | 53.3 |
| 4 | 9.0 | 12 | Parent | 34.4 | 25 | 30 | 20 | 25 | 34.4 | 28.3 |
| 4 | 10.6 | 30 | Child | 57.1 | 75 | 50 | 30 | 51.7 | 57.1 | 53.4 |
| 4 | 10.6 | 30 | Parent | 56.3 | 50 | 50 | 65 | 55 | 56.3 | 55.4 |
| 4 | 11.1 | 36 | Child | 56.3 | 60 | 45 | 55 | 53.3 | 56.3 | 54.4 |
| 4 | 11.1 | 36 | Parent | 28.1 | 45 | 35 | 60 | 46.7 | 28.1 | 40.2 |
| 5 | 10.6 | 0 | Child | 43.8 | 70 | 40 | 5 | 38.3 | 43.8 | 40.2 |
| 5 | 10.6 | 0 | Parent | 43.8 | 25 | 45 | 35 | 35 | 43.8 | 38.0 |
| 5 | 11.1 | 6 | Child | 31.3 | 45 | 45 | 0 | 30 | 31.3 | 30.4 |
| 5 | 11.1 | 6 | Parent | 56.3 | 25 | 37.5 | 30 | 30.8 | 56.3 | 39.7 |
| 5 | 11.6 | 12 | Child | 31.3 | 20 | 6.2 | 45 | 23.8 | 31.3 | 26.4 |
| 5 | 11.6 | 12 | Parent | 50.0 | 20 | 30 | 10 | 20 | 50 | 30.4 |
| 5 | 13.1 | 30 | Child | 43.8 | 39 | 30 | 20 | 26.7 | 43.8 | 32.6 |
| 5 | 13.1 | 30 | Parent | 46.9 | 25 | 25 | 20 | 23.3 | 46.9 | 31.5 |
| 5 | 13.6 | 36 | Child | 56.3 | 65 | 95 | 40 | 66.7 | 56.3 | 63.0 |
| 5 | 13.6 | 36 | Parent | 43.8 | 45 | 40 | 30 | 38.3 | 43.8 | 40.2 |
| 6 | 17.8 | 0 | Child | 53.1 | 80 | 40 | 30 | 50 | 53.1 | 51.1 |
| 6 | 17.8 | 0 | Parent | 46.9 | 45 | 40 | 25 | 36.7 | 46.9 | 40.2 |
| 7 | 13.7 | 0 | Child | 53.1 | 60 | 50 | 40 | 50 | 53.1 | 51.1 |
| 7 | 13.7 | 0 | Parent | 56.3 | 65 | 40 | 35 | 46.7 | 56.3 | 50 |

## Cognitive and neuropsychological assessment

##### Supplementary Table 4. Differential Ability Scales, Second Edition (DAS-II) scores at baseline for the cohort. The test was only performed at baseline.

|  | **Verbal** | **Nonverbal** | **Spatial** | **General Conceptual Ability Score** |
| --- | --- | --- | --- | --- |
| 1 | 65 | 79 | 69 | 68 |
| 2 | 81 | 69 | 68 | 70 |
| 3 | 33 | 37 | 34 | 40 |
| 4 | 60 | 69 | 75 | 65 |
| 5 | 73 | 78 | 86 | 76 |
| 7 | 86 | 91 | 80 | 83 |

##### Supplementary Table 5. Vineland Adaptive Behavior Scales (VABS-3) scores at baseline for the cohort. The test was only performed at baseline.

| **Patient ID** | **Communication** | **Daily Living Skills** | **Socialization** | **Adaptive Behavior Composite** |
| --- | --- | --- | --- | --- |
| 1 | 77 | 77 | 79 | 76 |
| 2 | 68 | 70 | 59 | 66 |
| 3 | 20 | 54 | 48 | 42 |
| 4 | 64 | 68 | 61 | 65 |
| 5 | 62 | 66 | 73 | 67 |
| 6 | 82 | 91 | 75 | 79 |
| 7 | 73 | 82 | 79 | 76 |
| 8 | 77 | 88 | 78 | 78 |
| 9 | 73 | 70 | 73 | 71 |

#####

## Ophthalmological assessment

## Cardiac assessment



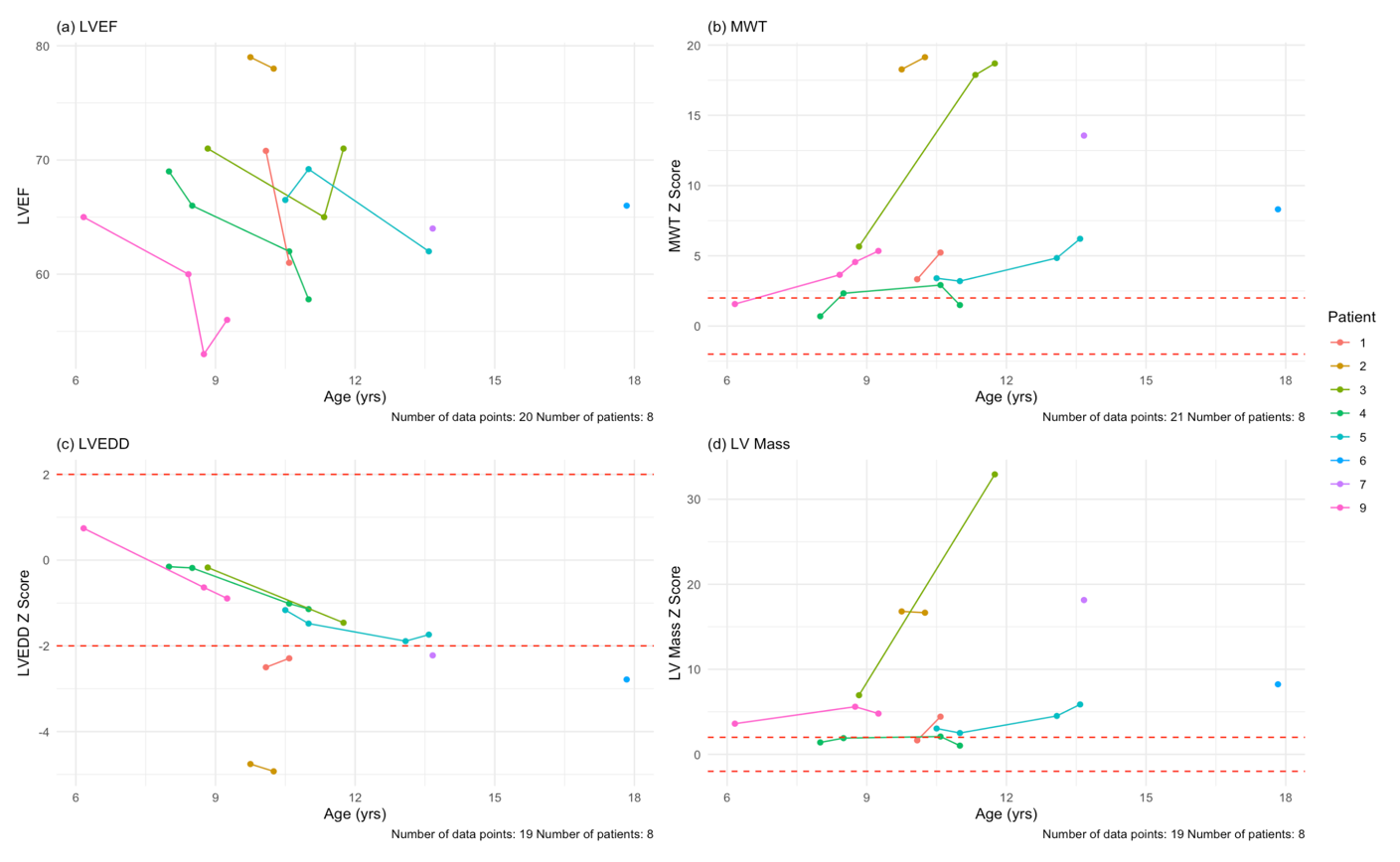


##### Supplementary Figure 1. These four graphs show all follow-up visits for cardiac measurements for each patient in the cohort. (a) Longitudinal changes in LVEF for the pediatric patients in the cohort. All patients with follow-up data had a decrease from their initial baseline LVEF. (b) Longitudinal changes in MWT z-scores for the pediatric patients in the cohort. Most exhibit myocardial thickening over time except for Patient 4, who experiences a mild decrease. (c) Longitudinal changes in LVEDD z-scores for the pediatric patients. (d) Longitudinal changes for LV mass for the pediatric patients. *MWT = maximum wall thickness, LVEDD = left ventricular end-diastolic dimension.*

## Pulmonary assessment

### Cardiopulmonary exercise testing


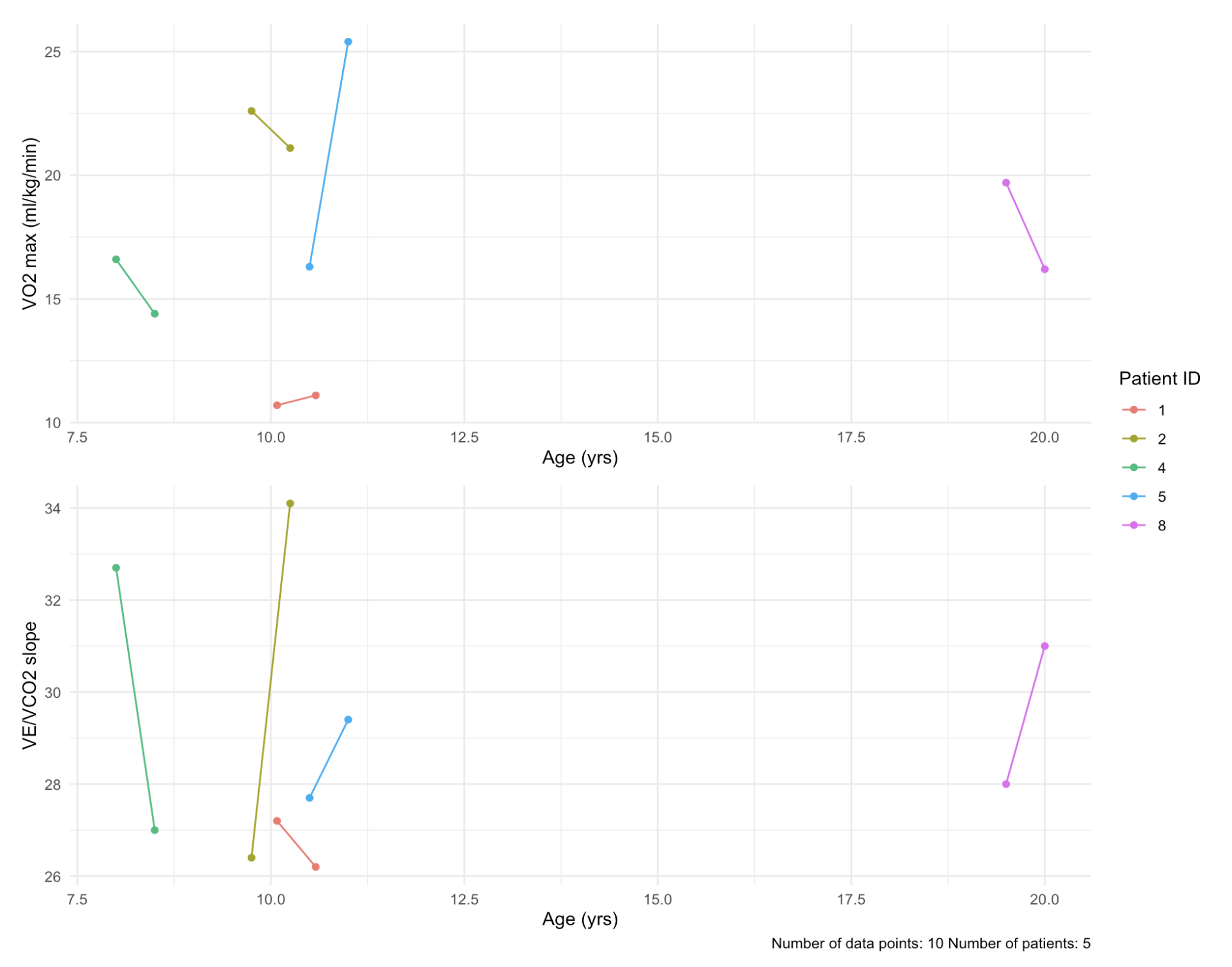


##### Supplementary Figure 2. (a) Longitudinal peak oxygen uptake (known as VO_2_ max) values for 5 patients in the cohort. (b) Longitudinal V_E_/VCO_2_ slope values plotted for 5 patients in the cohort.


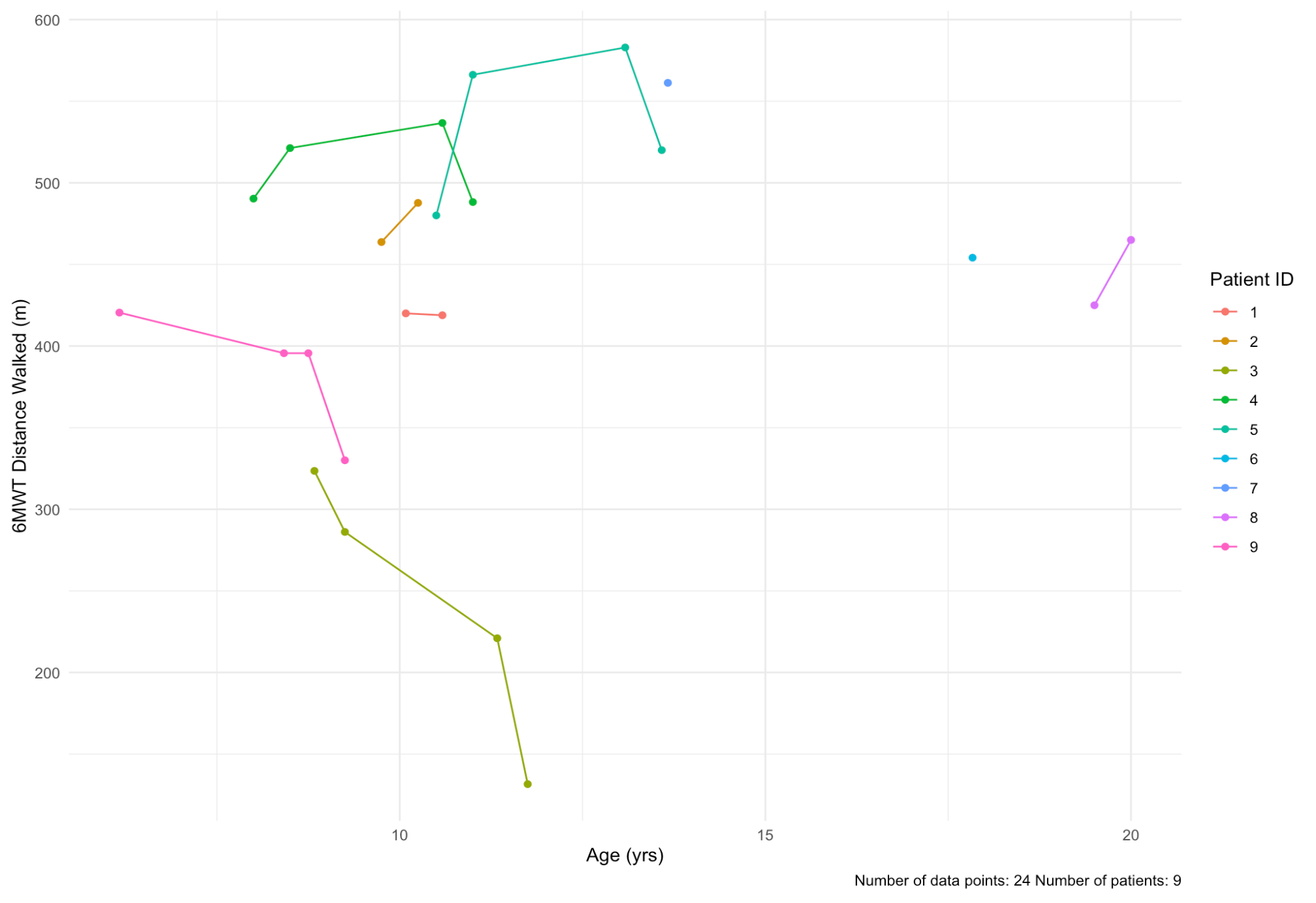


##### Supplementary Figure 3. Longitudinal data for all follow-up visits for the 6-minute walk test (6MWT) for each patient in the cohort. Patients 6 and 7 only had baseline data

##### Supplementary Table 6. Individual results of the CPET at baseline and at last follow-up. Mean follow-up time of 6 months. *CPET = cardiopulmonary exercise test, 6MWT = 6-minute walk test, SOB = shortness of breath, AT = anaerobic threshold, RQ = respiratory quotient.*

| **Patient ID** | **Age (yrs)** | **Exercise Time (min)** | **Reason for stopping** | **Peak VO_2_ max** | **% of Predicted VO2 max** | **V_E_/VCO_2_ slope** | **RQ** | **NYHA** |
| --- | --- | --- | --- | --- | --- | --- | --- | --- |
| 1 | 10.1 | 5.6 | Fatigue | 10.7 | 35 | 27.2 | 1 | 1 |
| 1 | 10.6 | 2.3 | Leg weakness | 11.1 | 38 | 26.2 | 0.92 | 1 |
| 2 | 9.8 | 6.2 | SOB | 22.6 | 45 | 26.4 | 0.88 | 1 |
| 2 | 10.3 | 15.1 | SOB | 21.1 | 42 | 34.1 | 1.05 | 2 |
| 4 | 8.0 | 6.6 | Fatigue | 16.6 | 28 | 32.7 | 0.83 | 2 |
| 4 | 8.5 | 7.3 | Leg weakness | 14.4 | 27 | 27 | 0.99 | 2 |
| 5 | 10.5 | 6.2 | SOB, leg weakness | 16.3 | 36 | 27.7 | 1.08 | 2 |
| 5 | 11.0 | 14.3 | SOB, leg weakness | 25.4 | 63 | 29.4 | 1.01 | 2 |
| 8 | 19.5 | 15.9 | SOB | 19.7 | 51 | 28.0 | 1.1 | 2 |
| 8 | 20.0 | 12.9 | SOB | 16.2 | 43 | 31.0 | 1.1 | 2 |

##### Supplementary Table 7. 6MWT results for all patients at baseline and at last-follow-up. Mean follow-up time was 23.2 months. *6MWT = 6-minute walk test.*

| **6MWT (distance walked, meters)** | | | | |
| --- | --- | --- | --- | --- |
|  | **Baseline** | | **Last Follow-Up (mean = 23.2 months)** | |
| **Patient ID** | **Age (yrs)** | **Distance (m)** | **Age (yrs)** | **Distance (m)** |
| 1 | 10.1 | 420 | 10.6 | 418.9 |
| 2 | 9.8 | 463.7 | 10.3 | 487.7 |
| 3 | 8.8 | 323.5 | 11.8 | 131.6 |
| 4 | 8.00 | 490.3 | 11.0 | 488.2 |
| 5 | 10.5 | 480 | 13.6 | 520 |
| 7 | 13.7 | 561.2 | 16.7 | 544.2 |
| 8 | 19.5 | 425.0 | 20.0 | 465 |
| 9 | 6.2 | 420.5 | 8.8 | 395.6 |

## Neuromuscular assessment

##### Supplementary Table 8. NSAA scores for each patient at different follow-up times. NSAA scores for the patients remained relatively stable during follow-ups. The maximum score is 34. *NSAA = North Star Ambulatory Assessment.*

| **Patient ID** | **Baseline** | **6 months** | **24 months** | **36 months** |
| --- | --- | --- | --- | --- |
| **1** | 25 | 25 | - | - |
| **2** | 29 | 29 | - | - |
| **3** | 7 | 13 | 8 | 4 |
| **4** | 34 | 34 | 34 | 34 |
| **5** | - | 33 | 33 | 34 |
| **6** | 32 | - | - | - |
| **7** | 34 | - | - | - |
| **8** | 34 | 34 | - | - |
| **9** | 29 | 33 | - | 33 |


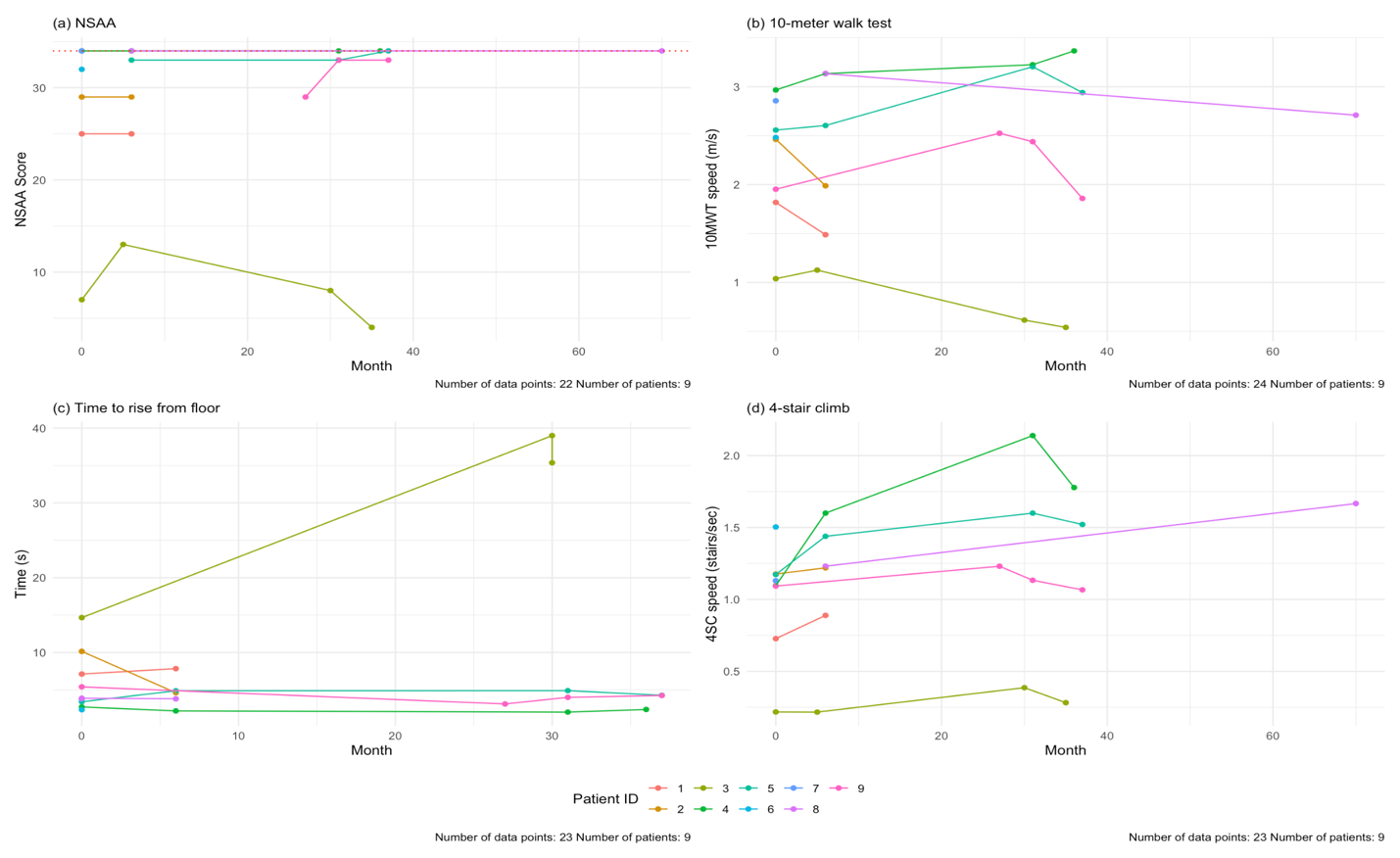


##### Supplementary Figure 4. All follow-up visits for: (a) NSAA scores. Patients 6 and 7 only had it performed at baseline. Refer to Table 8 for the values. The maximum score is 34 (red dotted line); (b) walking speed during the 10MWT for each patient; (c) 4SC speed for each patient. *NSAA = North Star Ambulatory Assessment, 10MWT = 10-minute walk test, 4SC = 4-stair climb test.*

## Laboratory parameters

##### Supplementary Table 9. Lab data for all patients at baseline and last follow-up. *AST = aspartate transaminase, ALT = alanine transaminase, BNP = brain natriuretic peptide, LDH = lactate dehydrogenase, CPK = creatine phosphokinase. * = age was 13.6 years, ^ = age was 8.5 years.*

| **Patient ID** | **Age (yrs)** | **Platelets (x10^9^/ L)** | **NT-proBNP (pg/mL)** | **AST (U/L)** | **ALT (U/L)** | **Creatinine (mg/dL)** | **Aldolase (U/L)** | **LDH (U/L)** | **CPK (U/L)** | **hs-cTnT (ng/L)** |
| --- | --- | --- | --- | --- | --- | --- | --- | --- | --- | --- |
| 1 | 10.1 | 276 | 492 | 348 | 325 | 0.37 | 24 | 947 | 1423 | 11 |
| 1 | 10.8 | 335 | 522 | 415 | 357 | 0.30 | 28.9 | 2322 | 1144 | 68 |
| 2 | 9.8 | 304 | 1791 | 407 | 280 | 0.52 | 15.6 | 1458 | 1004 | 11 |
| 2 | 11.6 | 292 | 3408 | 544 | 335 | 0.55 | 22.2 | 1412 | 1547 | 110 |
| 3 | 8.8 | 328 | 211 | 366 | 267 | 0.39 | 20.3 | 1261 | 858 | 11 |
| 3 | 11.8 | 328 | 3536 | 388 | 234 | 0.58 | 14.7 | 1458 | 719 | 109 |
| 4 | 8.0 | 292 | 89 | 206 | 131 | 0.58 | 16.4 | 635 | 526 | 11 |
| 4 | 11.0 | 270 | 140 | 213 | 133 | 0.60 | 12.5 | 630 | 533 | 14 |
| 5 | 10.5 | 291 | 194 | 693 | 192 | 0.51 | 16.4 | 904 | 1169 | 11 |
| 5 | 11.0 | 288 | 613 | 112 | 454 | 0.63 | 13.7 | 791 | 882 | 53* |
| 6 | 17.8 | 254 | 447 | 427 | 381 | 0.76 | 35.7 | 848 | 2840 | 31 |
| 6 | 18.2 | 292 | 714 | 390 | 383 | 0.80 | 33.9 | 828 | 2680 | 36 |
| 7 | 13.7 | 271 | 2520 | 573 | 430 | 0.73 | 31.2 | 1476 | 1744 | 99 |
| 8 | 19.5 | 278 | 2578 | 127 | 93 | 0.91 | 12.8 | 645 | 478 | 144 |
| 8 | 20.3 | 281 | 2888 | 120 | 95 | 1.08 | 10.3 | 590 | 440 | 171 |
| 9 | 6.5 | 316 | 143 | 516 | 238 | 0.53 | 24.5 | 1450 | 2502 | 20^ |
| 9 | 9.0 | 334 | 141 | 471 | 243 | 0.48 | 23.8 | 1395 | 2255 | 24 |


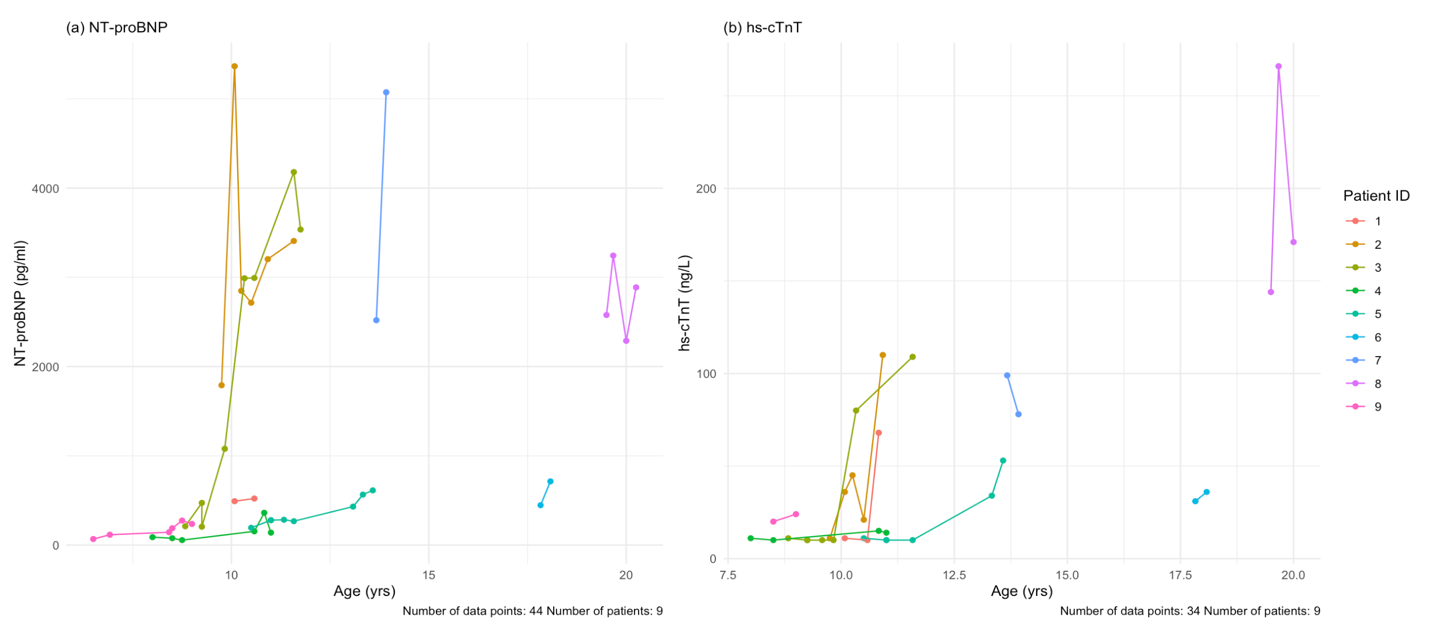


##### Supplementary Figure 5. (a) NT-proBNP values recorded for all visits for each patient in the study. (b) hs-cTnT values recorded for all visits for each patient in the study. *NT-proBNP = N-terminal pro brain natriuretic peptide, hs-cTnT = high sensitivity cardiac troponin T.*
